# Tirucallane Triterpenoids from the Stems and Stem Bark of *Cornus walteri* that Control Adipocyte and Osteoblast Differentiations

**DOI:** 10.3390/molecules23112732

**Published:** 2018-10-23

**Authors:** Seoung Rak Lee, Eunyong Choi, Se Hun Jeon, Xue Yan Zhi, Jae Sik Yu, Seon-Hee Kim, Jeongmi Lee, Ki-Moon Park, Ki Hyun Kim

**Affiliations:** 1 School of Pharmacy, Sungkyunkwan University, Suwon 16419, Korea; davidseoungrak@gmail.com (S.R.L.); jsyu@bu.edu (J.S.Y.); jlee0610@skku.edu (J.L.); 2 Sungkyun Biotech Co. LTD., Suwon 16419, Korea; eychoi8812@sungkyunbiotech.co.kr (E.C.); seonhee31@gmail.com (S.-H.K.); 3 School of Biotechnology and Bioengineering, Sungkyunkwan University, Suwon 16419, Korea; shjeon0507@gmail.com (S.H.J.); zhixueyan0214@gmail.com (X.Y.Z.); pkm1001@skku.edu (K.-M.P.)

**Keywords:** *Cornus walteri*, triterpenoid, cornusalterins N-P, mesenchymal stem cell, adipocytes, osteoblasts

## Abstract

*Cornus walteri* Wanger (Cornaceae) has been broadly used in traditional East Asian medicine for the treatment of various disorders, including skin inflammation and diarrhea. As part of our efforts to identify structurally and/or biologically new compounds from Korean medicinal plants, we have explored potentially new bioactive constituents from *C. walteri*. In the present study, seven triterpenoids (**1**–**7**) were isolated from *C. walteri* stems and stem bark. Compounds **1**–**3** were new tirucallane triterpenoids (cornusalterins N-P) and compounds **4**–**7** were isolated for the first time from *C. walteri*. The structures of the new compounds were determined based on 1D and 2D NMR spectroscopic data interpretations and HR-ESIMS, as well as a computational method coupled with a statistical procedure (DP4+). The regulatory effects of the isolated triterpenoids (**1**–**7**) on mesenchymal stem cell (MSC) differentiation to adipocytes and osteoblasts were examined in the C3H10T1/2 cell line. Although these compounds had little effect on MSC differentiation to osteoblasts, lipid droplet formation in adipocyte-differentiated MSCs decreased in the presence of the seven triterpenoids. Compounds **1** and **4** each had a relatively distinct correlation between dose and efficacy, showing adipogenesis suppression at higher concentrations. Our findings demonstrate that the active compounds **1** and **4** can exert beneficial effects in regulation of adipocyte differentiation.

## 1. Introduction

*Cornus* is a genus comprised of approximately 30–60 species of woody plants in the family Cornaceae, which is generally identified using morphological features, including berries, blossoms, and bark [1,2]. *Cornus walteri* Wanger (Cornaceae) grows in mountain valleys and is distributed in eastern Asia and China [3]. The fruits and leaves of *C. walteri* have been widely used in China to treat skin inflammation, and the leaves have been used in Korean traditional medicine to alleviate diarrheal symptoms [3,4,5]. Previous phytochemical investigations of this plant resulted in the isolation of various triterpenoids, δ-valerolactones, and flavonoids [4,5,6,7]. The extracts of *C. walteri* have been investigated for a variety of biological effects, including anti-hyperglycemia, anti-inflammation, and anti-obesity [8,9]. 

**Figure 1:**
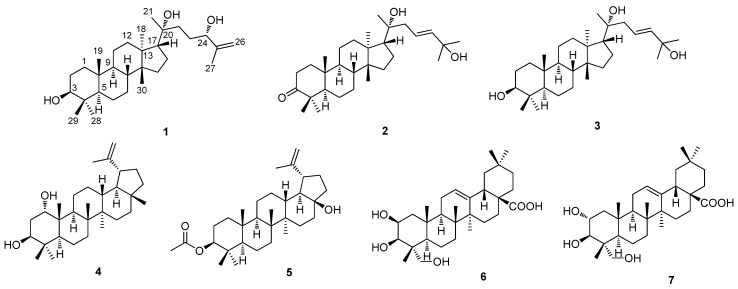
Chemical structures of compounds **1**–**7** from *C. walteri*.

In our efforts to identify structurally and/or biologically new compounds from Korean medicinal plants [10,11,12,13,14], we have explored potentially new bioactive constituents from *C. walteri* [4,6,7,12]. Our previous research had identified naturally-occurring triterpenoids from MeOH extracts of *C. walteri* stems and stem bark, which included new tirucallane-type triterpenoids with cytotoxic effects towards A549, SK-OV-3, and SK-MEL-2, and lupane triterpenoids with a protective effect against cisplatin-induced nephrotoxicity [4,7,12]. New cytotoxic δ-valerolactones were also identified from MeOH extracts in our previous study [6]. These findings led us to further investigate potential bioactive constituents from the MeOH extracts. Therefore, we conducted additional phytochemical analysis of *C. walteri* MeOH extracts, which led to the isolation of seven triterpenoids, including three new tirucallane triterpenoids, cornusalterins N-P (**1**–**3**) (Figure 1). Here, we describe the isolation, structural elucidation of the compounds (**1**–**7**) and their potential for regulating adipocyte and osteoblast differentiation.

## 2. Results and Discussion

### 2.1. Chemical Identification of the Isolated Compounds from C. walteri

**Figure 2:**
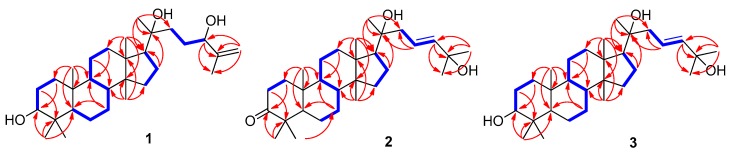
Key COSY (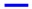) and HMBC (→) correlations for compounds **1**–**3**.

**Figure 3:**
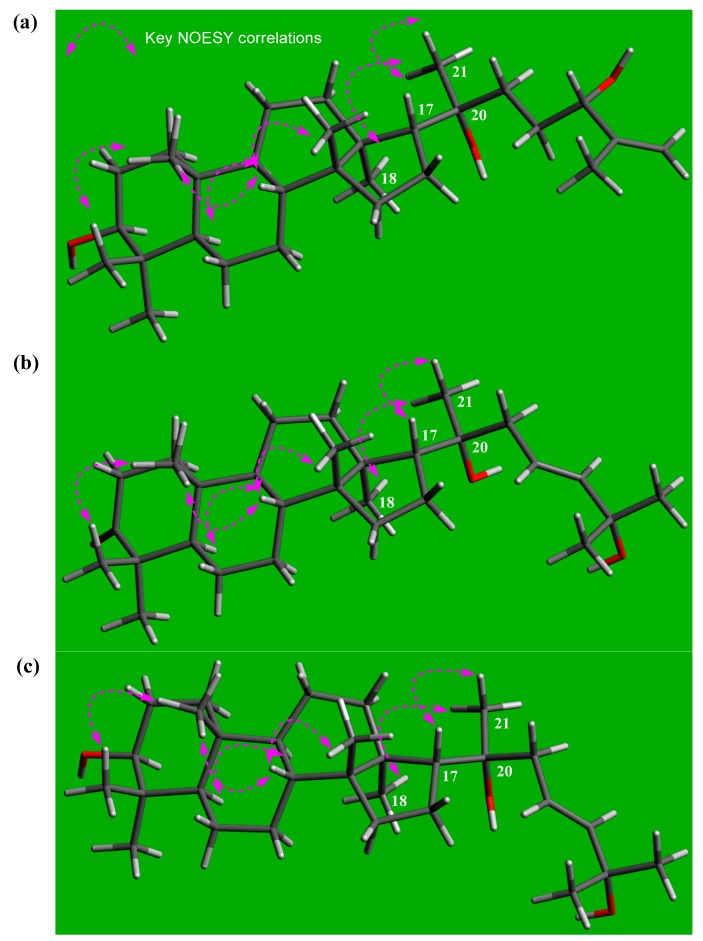
Important NOESY correlations of compounds **1**–**3**. (**a**) cornusalterin N (**1**); (**b**) cornusalterin O (**2**); and (**c**) cornusalterin P (**3**).

Cornusalterin N (**1**) was purified as a white amorphous powder and its molecular formula of C_30_H_52_O_3_ was determined based on the positive-ion mode high resolution electrospray ionization (HR-ESI)-MS data at *m*/*z* 483.3815 [M + Na]^+^ (Calcd for C_30_H_52_O_3_Na, 483.3809). The IR spectrum of **1** showed two functional groups, including hydroxy groups (3595 cm^−1^) and a double bond (1689 cm^−1^). The ^1^H and ^13^C NMR spectroscopic data (Table 1) of **1** were similar to the NMR data of cornusalterin M, which had been previously identified from *C. walteri* by our group [12]. There was one exception between compound **1** and cornusalterin M, which was the presence of chemical shift (*δ*_H_ 3.22; *δ*_C_ 78.9) of a hydroxylated methine in **1**, as opposed to the ketone moiety value (*δ*_C_ 220.0) of C-3 observed in cornusalterin M [12]. Detailed analysis of the ^1^H-^1^H COSY, HMQC, and HMBC spectra of **1** revealed the complete gross structure (Figure 2). In particular, the location of a hydroxy group at C-3 was unambiguously confirmed by key HMBC correlations of H-5, H_3_-28, and H_3_-29 to C-3. Also, a proton spin-spin coupling system from H_2_-1 to H-3 in the ^1^H-^1^H COSY spectrum supported a hydroxy group at C-3. A hydroxy group at C-20 was identified based on the distinctive carbon chemical shift of C-20 (*δ*_C_ 75.4) and long-range correlations in HMBC from H_2_-16, H-17, H_3_-21, H_2_-22, and H_2_-23 to C-20. In addition, HMBC correlations of H_2_-26/C-24 and H_3_-27/C-24 provided evidence of a hydroxy group at C-24 and a Δ^25,26^-double bond. The relative configuration of **1** was determined by analyzing NOESY data where the *α*-oriented position of the hydroxy group at C-20 was assigned by NOESY correlations of H-17/H_3_-21 and H_3_-18/H_3_-21 (Figure 3) [12,15]. The absolute configurations of C-20 and C-24 were achieved by the gauge-including atomic orbital (GIAO) NMR chemical shifts calculation, which could be followed by DP4+ calculations [16]. The calculated ^13^C NMR chemical shifts of four possible diastereomers **1a** (20*R*,24*S*), **1b** (20*S*,24*S*), **1c** (20*R*,24*R*), and **1d** (20*S*,24*R*) were compared with the experimental values of **1** by utilizing DP4+ probability analysis. The statistical results indicated the structural equivalence of **1** to **1a** (20*R*,24*S*) with 98.37% probability (Supplementary Materials). Thus, the structure of **1** is shown in Figure 1.

Cornusalterin O (**2**), isolated as a white amorphous powder, has a molecular formula of C_30_H_50_O_3_, which was determined by the positive-ion mode HR-ESI-MS data at *m*/*z* 481.3657 [M + Na]^+^ (Calcd for C_30_H_50_O_3_Na, 481.3652). Evaluation of the ^1^H and ^13^C NMR data of **2** suggested that the NMR spectroscopic values were nearly identical to values of cornusalterin B. This indicated that compound **2** was an analogue of a tirucallane-type triterpenoid [7,12]. A comparison of the NMR data of **2** with that of cornusalterin B indicated that the chemical shift (*δ*_C_ 75.2) of C-20 in **2** was shifted as compared to (*δ*_C_ 37.7) C-20 in cornusalterin B and that the signals for a methoxy group [*δ*_H_ 3.16 (3H, s); *δ*_C_ 50.4] at C-25 in cornusalterin B were absent in **2** [7]. The hydroxy group at C-20 was unambiguously identified by key HMBC correlations of H_2_-16/C-20, H-17/C-20, H_3_-21/C-20, and H_2_-23/C-20 (Figure 2) and NOESY correlations of H-17/H_3_-21 and H_3_-18/H_3_-21. These results led to the assignment of an *α*-oriented position of the hydroxy group at C-20 (Figure 3) [12,15]. Also, key HMBC correlations of H-23, H-24, H_3_-26, and H_3_-27 to C-25 suggested that an additional hydroxy group was located at C-25, which was also confirmed by a carbon chemical shift (*δ*_C_ 71.0) at C-25. The stereochemistry of **2** was determined by analyzing the NOESY data and had the same stereochemistry of **1**. In addition, DP4+ analysis was carried out to determine the absolute configuration at C-20. The calculated ^13^C NMR chemical shifts of two possible isomers **2a** (20*R*) and **2b** (20*S*) were subjected to DP4+ analysis with the experimental values, which indicated that isomer **2a** (20*R*) shows a DP4+ probability score of 100% (Supplementary Materials). Accordingly, the structure of **2** is shown in Figure 1.

The positive-ion mode HR-ESI-MS data at *m*/*z* 483.3812 [M + Na]^+^ (Calcd for C_30_H_52_O_3_Na, 483.3809) of cornusalterin P (**3**), which was isolated as a white amorphous powder, had a molecular formula of C_30_H_52_O_3_. Using 1D and 2D NMR data from **3**, we unambiguously determined that the structure of **3** was nearly identical to **2**. However, an oxygenated methine at C-3 [*δ*_H_ 3.20 (1H, dd, *J* = 11.5, 5.0 Hz); *δ*_C_ 79.1] in **3** replaced the ketone moiety (*δ*_C_ 218.5) at C-3 in **2**. Key HMBC correlations of H_2_-1, H-5, H_3_-28, and H_3_-29 to C-3 allowed us to assign the location of a hydroxy group at C-3. This result was also confirmed via a proton spin-spin coupling system from H_2_-1 to H-3 in the ^1^H-^1^H COSY data (Figure 2). The relative configuration of **3** was identical to that of **2**, which was determined via the NOESY data of **3** (Figure 3). To verify the absolute configuration of C-20, the DP4+ protocol was again applied to the simulated ^13^C NMR chemical shifts of the two possible isomers **3a** (20*R*) and **3b** (20*S*). The results showed that isomer **3a** (20*R*) was the correct structure for **3**, with 99.98% probability (Supplementary Materials). Therefore, the structure of **3** is shown in Figure 1. 

By comparing NMR spectroscopic data with previously reported data, the other isolated compounds were identified as glochilocudiol (**4**) [17], 3*β*-acetoxy-28-norlup-20(29)-en-17*β-*ol (**5**) [18], bayogenin (**6**) [19], and arjunolic acid (**7**) [20]. Although **4**–**7** have been previously reported, **4**–**7** were isolated and identified for the first time from *C. walteri*.

### 2.2. Regulatory Effects of the Compounds on Mesenchymal Stem Cell Differentiation into Adipocytes and Osteoblasts

Mesenchymal stem cells (MSCs) differentiate into various cells, including adipocytes and osteoblasts [21,22]. As aging progresses, changes in the internal and external determinants were involved in the differentiation of mesenchymal stem cells into adipocytes and osteoblasts [23,24]. Aging continues to reduce bone mass and to increase fat cells in the bone marrow. In the bone marrow stromal cells, adipocyte differentiation and osteoblast differentiation are inversely correlated, which can promote osteogenesis when adipocyte differentiation is inhibited [25,26]. The C3H10T1/2 cell line, which originates from mouse embryonic fibroblasts, is a multipotent stem cell line that can differentiate into various cell lines, including osteoblasts and adipocytes. C3H10T1/2 cell lines have been used in various studies to regulate the differentiation of progenitor cells [27,28].

**Figure 4:**
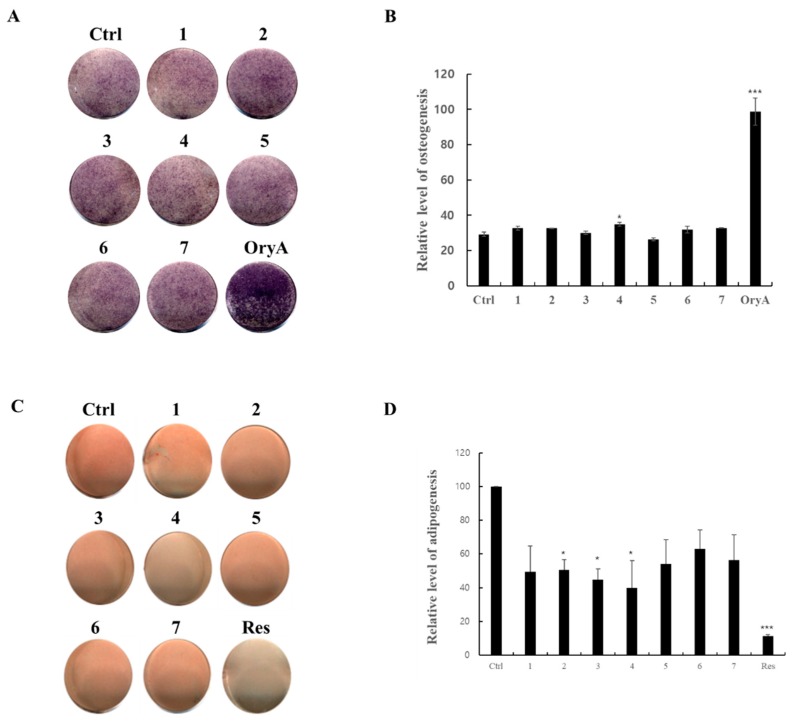
The effects of compounds **1**–**7** on the differentiation of MSCs toward osteoblasts or adipocytes. The mouse mesenchymal stem cell line, C3H10T1/2, was treated with compounds **1**–**7**. After osteogenic differentiation, the cells were stained with ALP (**A**) and ALP enzyme activity was measured (**B**). In the separate plates, the cells were differentiated into adipocytes prior to ORO staining (**C**). Stained cells were quantitatively evaluated by resolving stained lipid droplets and measuring absorbance at the red wavelength (**D**). Ctrl represents untreated negative control. 5 μM of oryzativol A (OryA) was added to the experimental set as a positive osteogenesis control. 20 μM of resveratrol (Res) was used as a positive control in adipogenesis. 10 μM of each of the compounds was added to the osteogenesis- or adipogenesis-differentiation medium. * denotes *p* < 0.05 and *** denotes *p* < 0.001.

In order to evaluate the effects of the isolated triterpenoids (**1**–**7**) on early stages of osteoblast differentiation, each compound was added to the MSC culture media during osteogenesis. Cells were stained for alkaline phosphatase (ALP) expression 10 days after the onset of osteogenesis (Figure 4A,B). The staining intensities of the compound-treated cells did not differ from that of the untreated, negative control cells. Although **4** tended to induce slightly higher levels of ALP activity compared to the untreated control, all tested compounds failed to show a significant induction of ALP expression. These results have demonstrated that none of the compounds affect ALP activity or osteogenesis in MSC differentiation. At the same time, C3H10T1/2 cell lines were induced into adipogenic differentiation. During adipogenic differentiation, 10 μM of each compound was added to the MSC culture media. After adipogenic differentiation for nine days, cells were stained with Oil Red O (Figure 4C). All the isolated triterpenoids (**1**–**7**) slightly inhibited adipocyte differentiation with 40~60% suppression compared to non-treated negative control (Figure 4D). Therefore, all compounds were further tested for evaluation of suppressive effect on adipogenesis.

**Figure 5:**
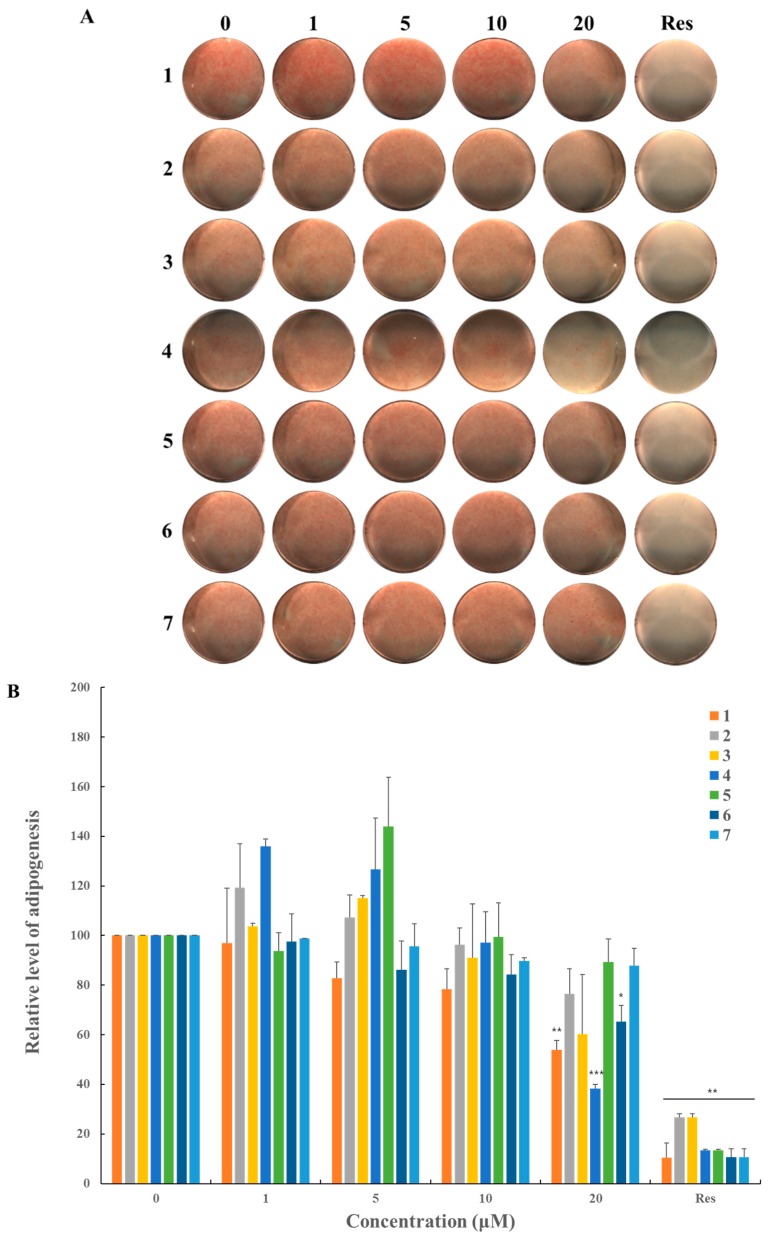

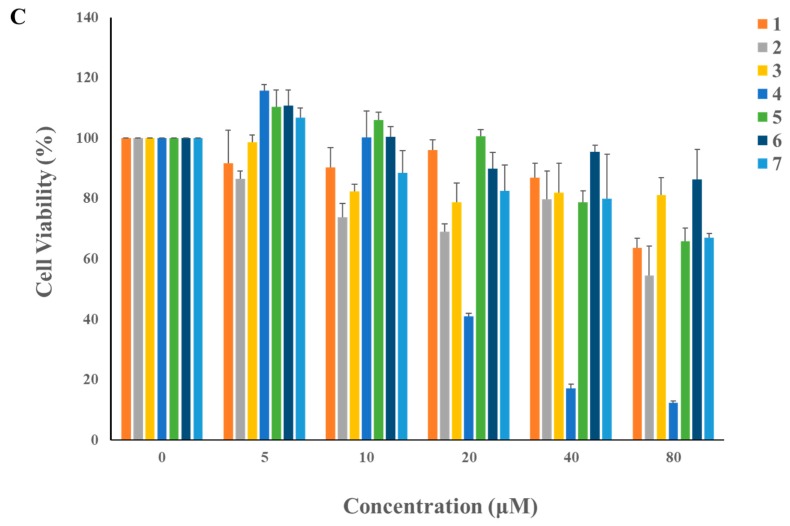
Suppressive effects of compounds **1**–**7** on adipogenic differentiation. C3H10T1/2 cells were treated with sequential concentrations (1, 5, 10, and 20 μM) of the compounds for nine days prior to ORO staining. (**A**) Stained cells were quantitatively evaluated by resolving stained lipid droplets and measuring absorbance at the red wavelength; (**B**) 20 μM of resveratrol (denoted as Res) was used as the positive control. The values were relatively calculated by setting the untreated negative control to 100. C3H10T1/2 cells were treated with higher concentrations (5, 10, 20, 40, and 80 μM) of the compounds to evaluate the cellular toxicity; (**C**) Cell viability was calculated relatively by setting the untreated negative control to 100. * denotes 0.01 < *p* < 0.05, ** denotes 0.001 < *p* < 0.01, and *** denotes *p* < 0.001.

The isolated triterpenoids marginally inhibited lipid formation in MSCs at levels comparable to the positive control, 20 μM resveratrol (Figure 5A,B). Various concentrations of the compounds were tested in lipid droplet production during adipogenesis of the MSCs. After day nine of adipogenic differentiation, cells were treated with Oil Red O (ORO) stain, and the staining was quantified by resolving in iso-propanol. All of the compounds suppressed formation of lipid droplets in a dose-dependent manner. With 20 μM of each respective compound, the treated cells showed 40–60% inhibition of adipocyte differentiation compared to the untreated negative control. Even at the highest compound concentrations, none of the compounds showed an effect on MSC differentiation as high as the 20 μM resveratrol positive control. Among the compounds, compounds **1** and **4** showed relative correlations between dose and efficacy and suppression of adipogenesis at higher concentrations. Although none of the triterpenoids were superior to resveratrol, it is expected that the combined activity of all the compounds will be greater than each individually. However, compound **4** induced cellular toxicity from the concentration of 20 μM showing 40% cell viability compared to the non-treated control (Figure 5C). Regarding to the cytotoxicity, although **4** inhibits formation of lipid droplet in differentiated adipocytes, this cellular toxicity may influence the adipogenic differentiation and adipocyte proliferation in some parts. In contrast to **4**, compound **1** inhibited adipocyte differentiation without any severe cellular toxicity in MSC cells.

## 3. Materials and Methods

### 3.1. General Experimental Procedures

Optical rotations were acquired on a Jasco P-1020 polarimeter. IR spectra were obtained on a Bruker IFS-66/S FT-IR spectrometer. ESI and HR-ESI mass spectra were measured on a SI-2/LCQ DecaXP Liquid chromatography (LC)-mass spectrometer. NMR spectra, including ^1^H-^1^H COSY, HMQC, HMBC, and NOESY experiments, were recorded on a Varian UNITY INOVA 500 NMR spectrometer operating at 500 MHz (^1^H) and 125 MHz (^13^C). Chemical shifts are given in ppm (δ). Preparative high-performance liquid chromatography (HPLC) was performed using a Gilson 306 pump with a Shodex refractive index detector. Silica gel 60 (Merck, Darmstadt, Germany, 230–400 mesh) and RP-C18 silica gel (Merck, 230–400 mesh) were used for column chromatography. Merck precoated silica gel F254 plates and RP-18 F254s plates were used for thin layer chromatography (TLC). Spots were detected on TLC under UV light or by heating after the spots were sprayed with anisaldehyde-sulfuric acid.

### 3.2. Plant Material

*C. walteri* stems and stem bark were obtained from Jeju Island, Korea, in October 2014. The plants were identified by one of authors (K. H. K). A voucher specimen (SKKU MC-2014) was deposited in the herbarium of the School of Pharmacy, Sungkyunkwan University, Suwon, Korea.

### 3.3. Extraction and Isolation

*C. walteri* stems and stem bark (3.0 kg) were dried, chopped, extracted with 80% MeOH (5 L × 3) at room temperature, and then filtered. The filtered 80% MeOH extract was concentrated in vacuo to acquire a MeOH extract (310 g). The MeOH extract was dissolved in distilled water (6.5 L) and successively solvent-partitioned using hexane, CHCl_3_, and *n*-BuOH (700 mL × 3), which resulted in 15.0, 32.0, and 45.0 g of each fraction, respectively. The hexane-soluble fraction (15.0 g) was fractionated by silica gel column chromatography (230–400 mesh) and eluted with a gradient solvent system of hexane-EtOAc (5:1 to 1:1, *v*/*v*) to obtain five fractions (H1–H5). Fraction H1 (4.0 g) was separated by a RP-C_18_ silica gel (230–400 mesh) column using 100% MeOH to acquire five subfractions (H11–H15). The fraction H13 (950 mg) was added to a silica gel (230–400 mesh) column and eluted with hexane-EtOAc (7:1, *v*/*v*). The fraction was successively purified using semi-preparative reverse-phase HPLC (Econosil C18 column, 250 × 10.0 mm, 5 μm, flow rate: 2 mL/min) with 100% MeOH. From this purification, we obtained compound **5** (6 mg). The fraction H4 (1.5 g) was subjected to a RP-C_18_ silica gel (230–400 mesh) column and eluted with 100% MeOH, which resulted in six subfractions (H41–H46). The fraction H41 (190 mg) was passed over a Sephadex-LH20 column with 100% MeOH. The fraction was successively isolated by semi-preparative reverse-phase HPLC (Econosil C18 column, 250 × 10.0 mm, 5 μm, flow rate: 2 mL/min) using an isocratic solvent system of 90% MeOH. From this step we obtained compound **1** (5 mg). The fraction H42 (250 mg) was separated by a silica gel (230–400 mesh) column with an isocratic solvent system of hexane-EtOAc (3:1, *v*/*v*) to obtain three subfractions (H421–H423). Compound **4** (5 mg) was purified from H422 (45 mg) by semi-preparative normal-phase HPLC (Apollo Silica column, 250 × 10.0 mm, 5 μm, flow rate: 2 mL/min) with an isocratic solvent system of hexane-EtOAc (3:1, *v*/*v*). The fraction H5 (700 mg) was passed through a RP-C_18_ silica gel (230–400 mesh) column and then fractionated with 85% MeOH to provide five subfractions (H51–H55). Compounds **6** (8 mg) and **7** (10 mg) were isolated from the subfraction H53 (60 mg) using semi-preparative normal-phase HPLC (Apollo Silica column, 250 × 10.0 mm, 5 μm, flow rate: 2 mL/min) with an isocratic solvent system of CHCl_3_-MeOH (17:1, *v*/*v*). The fraction H54 (150 mg) was separated by using semi-preparative normal-phase HPLC (Apollo Silica column, 250 × 10.0 mm, 5 μm, flow rate: 2 mL/min) and eluted with an isocratic solvent system of CHCl_3_-MeOH (22:1, *v*/*v*), resulting in compounds **2** (5 mg) and **3** (10 mg).

#### 3.3.1. Cornusalterin N (**1**)

White amorphous powder; ${\left[\alpha \right]}_{D}^{25}$-25.9 (*c* 0.35, CHCl_3_); IR (KBr) *v*_max_ 3595, 2942, 1689, 1377, 725 cm^−1^; HR-ESIMS *m*/*z* 483.3815 [M + Na]^+^ (Calcd for C_30_H_52_O_3_Na, 483.3809).

#### 3.3.2. Cornusalterin O (**2**)

White amorphous powder; ${\left[\alpha \right]}_{D}^{25}$-14.3 (*c* 0.20, CHCl_3_); IR (KBr) *v*_max_ 3581, 2934, 2840, 1691, 1388, 721 cm^−1^; HR-ESIMS *m*/*z m*/*z* 481.3657 [M + Na]^+^ (Calcd for C_30_H_50_O_3_Na, 481.3652).

#### 3.3.3. Cornusalterin P (**3**)

White amorphous powder; ${\left[\alpha \right]}_{D}^{25}$-7.9 (*c* 0.15, CHCl_3_); IR (KBr) *v*_max_ 3589, 2938, 2831, 1697, 1354, 743 cm^−1^; HR-ESIMS *m*/*z* 483.3812 [M + Na]^+^ (Calcd for C_30_H_52_O_3_Na, 483.3809).

### 3.4. Computational Analysis

Conformational searches were performed using the Tmolex 4.3.1 with the DFT settings (B3-LYP functional/M3 grid size), geometry optimization settings (energy 10^−6^ hartree, gradient norm |d*E*/d*xyz*| = 10^−3^ hartree/bohr), and the basis set def-SV(P) for all atoms. NMR shielding constants calculations were performed on the optimized ground state geometries at the DFT B3LYP/def-SV(P) level of theory. The NMR chemical shifts of the isomers were obtained by Boltzmann averaging the ^13^C NMR chemical shift of the stable conformers at 298.15 K. Chemical shift values were calculated using the equation below where $\delta_{calc}^{x}$ is the calculated NMR chemical shift for nucleus *x*, and $\sigma^{o}$ is the shielding tensor for the proton and carbon nuclei in tetramethylsilane calculated at the DFT B3LYP/def-SV(P) basis set [29].

$$\;\delta_{calc}^{x}\;=\;\frac{\sigma^{o}-\;\sigma^{x}}{1-{\;\sigma }^{o}/10^{6}}\;$$

The calculated NMR properties of optimized structures were averaged based upon their respective Boltzmann populations and calculations of DP4+ probability analysis were facilitated by the Excel sheet (DP4+) provided by Grimblat et al. [16]. 

### 3.5. Cell Culture and Differentiation

The C3H10T1/2 cell line was purchased from American Type Culture Collection (ATCC, Manassas, VA, USA). Cells were grown in Dulbecco’s modified Eagle’s medium (DMEM, Hyclone, Laboratories Inc., Logan, UT, USA) containing 10% fetal bovine serum (Hyclone) and antibiotics (100 U/mL of penicillin and 100 μg/mL of streptomycin, Hyclone) at 37 °C in a humidified atmosphere of 5% CO_2_.

For adipogenic differentiation, cells were grown to confluence. Two days after confluence, the medium was replaced with fresh DMEM (10% FBS, 100 U/mL of penicillin, and 100 µg/mL of streptomycin) supplemented with 1 µM dexamethasone (Sigma-Aldrich, St. Louis, MO, USA), 10 µM troglitazone (Sigma-Aldrich), and 5 μg/mL insulin (Sigma-Aldrich), 0.5 mM IBMX (3-isobutyl-1-methylxanthine, Sigma-Aldrich). Seventy-two hours later, the medium was replaced with DMEM (10% FBS, 100 U/mL of penicillin, and 100 µg/mL of streptomycin), 1 µM dexamethasone, 5 μg/mL insulin. During the 3–5 days of growth, the media was replaced every three days. For osteogenic differentiation, cells that reached confluence were cultured with DMEM (10% FBS, 100 U/mL of penicillin, and 100 µg/mL of streptomycin), 10 mM β-glycerophosphate (Sigma-Aldrich), and 50 μg/mL ascorbic acid (Sigma-Aldrich) for 9–12 days. The medium was replaced every three days.

### 3.6. Alkaline Phosphatase (ALP) Staining

At 9–12 days after differentiation, the medium was removed for ALP staining. The cells were washed twice with 2 mM MgCl_2_, and then cells were immersed in AP buffer (100 mM Tris−HCl, pH 9.5, 100 mM NaCl, and 10 mM MgCl_2_) for 15 min. The cells were then incubated in AP buffer containing 0.4 mg/mL nitro-blue tetrazolium (NBT, Sigma) and 0.2 mg/mL 5-bromo-4-chloro-3-indolyl phosphate (BCIP, Sigma). The reaction was stopped with a 5 mM EDTA solution (pH 8.0). The cells were fixed and washed with water twice.

### 3.7. Quantification of Alkaline Phosphatase (ALP) Activity

ALP activity was quantified using an Alkaline Phosphatase Assay Kit (ab83369; Abcam, Cambridge, MA, USA). Cell lysates were collected according to the manufacturer’s recommendations. Each sample was incubated in the dark with a *p*-nitrophenyl phosphate solution in 96-well plates at 25 °C for 60 min. To stop the reaction, stop solution was added to each well, and the optical density was measured at 405 nm.

### 3.8. Oil Red O (ORO) Staining

After adipogenic differentiation, cells fixed with 10% formaldehyde solution (Sigma-Aldrich) were stained with 0.5% Oil Red O solution (Sigma-Aldrich) for 1 h. Cells were washed with distilled water three times to stop the reaction. To quantify intra-cellular triglyceride content, stained cells were dissolved in 1 mL of isopropyl alcohol, and the absorbance was measured at 520 nm using a SpectraMax M2/M2e Microplate Reader (Molecular Devices, Sunnyvale, CA, USA).

### 3.9. Cell Viability

Cell viability was determined using the 3-(4,5-dimethylthiazol-2-yl)-2,5-diphenyl-tetrazolium-bromide (MTT) assay [30,31,32]. C3H10T1/2 cells were seeded at 1.5 × 10^4^ cells per well and were cultured until reaching confluence. The cells were treated with 5, 10, 20, 40, and 80 μM of each compound. After 48 h, MTT (5 mg/mL in PBS) was added, and the cells were incubated at 37 °C for an additional 4 h. The formazan crystals were dissolved in 200 μL DMSO, and the absorbance was measured at 520 nm using a microplate reader.

## 4. Conclusions

In the present study, phytochemical analysis of the MeOH extract of *C. walteri* stems and stem bark led to the isolation of new tirucallane triterpenoids (**1**–**3**), cornusalterins N-P along with four known tirucallane triterpenoids (**4**–**7**), which were isolated for the first time from *C. walteri*. All the isolated compounds were evaluated for their regulatory effects on MSC differentiation to adipocytes and osteoblasts in the C3H10T1/2 cell line. Compounds **1** and **4** suppressed formation of lipid droplets in a dose-dependent manner, suggesting the inhibition of adipocyte differentiation, while none of the isolated compound showed the induction of ALP expression. These findings provide experimental evidence for the anti-adipogenic property of *C. walteri* and support the potential that **1** and **4** can exert beneficial effects in regulation of adipocyte differentiation.

## Figures and Tables

**Table 1:** ^1^H (500 MHz) and ^13^C (125 MHz) NMR data for compounds **1**–**3** in CDCl_3_. ^a^

Position	1		2		3	
	*δ*_H_	*δ*_C_	*δ*_H_	*δ*_C_	*δ*_H_	*δ*_C_
1	1.64 m 1.15 m	39.2 t	1.76 m 1.28 m	40.1 t	1.69 m 1.07 m	39.3 t
2	1.75 m 1.65 m	27.5 t	2.51 m, 2.43 m	34.3 t	1.82 m, 1.64 m	27.6 t
3	3.22 dd (11.5 5.0)	78.9 d		218.5 s	3.20 dd (11.5, 5.0)	79.1 d
4		38.9 s		47.6 s		39.2 s
5	0.75 m	55.8 d	1.36 m	55.6 d	0.73 m	56.1 d
6	1.72 m 1.50 m	18.3 t	1.75 m, 1.53 m	22.2 t	1.70 m, 1.48 m	18.5 t
7	1.52 m, 1.28 m	24.8 t	1.52 m, 1.30 m	25.0 t	1.51 m, 1.28 m	25.0 t
8	1.43 m	42.4 d	1.41 m	42.7 d	1.40 m	42.6 d
9	1.34 m	50.6 d	1.35 m	50.2 d	1.32 m	50.9 d
10		37.1 s		37.0 s		37.4 s
11	1.53 m, 1.06 m	21.5 t	1.46 m, 1.09 m	19.9 t	1.45 m, 1.07 m	21.7 t
12	1.74 m, 1.22 m	25.4 t	1.71 m, 1.23 m	25.1 t	1.73 m, 1.21 m	25.1 t
13		40.3 s		40.5 s		40.6 s
14		50.3 s		50.4 s		50.5 s
15	1.52 m, 1.29 m	31.2 t	1.56 m, 1.30 m	31.3 t	1.49 m, 1.27 m	31.3 t
16	1.82 m, 1.21 m	27.4 t	1.86 m, 1.23 m	27.7 t	1.84 m, 1.25 m	27.5 t
17	1.49 m	50.1 d	1.74, m	50.1 d	1.72 m	50.1 d
18	0.97 s	15.5 q	1.00 s	15.4 q	0.96 s	15.7 q
19	0.86 s	16.2 q	0.95 s	16.2 q	0.85 s	16.4 q
20		75.4 s		75.2 s		75.3 s
21	1.16 s	25.3 q	1.14 s	26.1 q	1.13 s	26.0 q
22	1.42 m	36.3 t	2.21 m	43.6 t	2.20 m	43.6 t
23	1.83 m, 1.61 m	29.3 t	5.70 m	122.5 d	5.70 m	122.6 d
24	4.06 t (6.0)	76.2 d	5.71 m	142.3 d	5.71 m	142.2 d
25		147.6 s		71.0 s		71.0 s
26	4.96 br s, 4.86 br s	110.7 t	1.33 s	30.2 q	1.33 s	30.2 q
27	1.75 s	18.0 q	1.33 s	30.1 q	1.33 s	30.1 q
28	0.99 s	28.0 q	1.08 s	27.0 q	0.97 s	28.2 q
29	0.79 s	15.3 q	1.04 s	21.2 q	0.77 s	15.6 q
30	0.89 s	16.4 q	0.88 s	16.5 q	0.87 s	16.6 q

^a^*J* values are in parentheses and reported in Hz.

## Supplementary Materials

1D and 2D NMR data, computational and statistical data of **1**–**3** are available free of charge on the Internet.
