# Supplementary material for: Tirucallane Triterpenoids from the Stems and Stem Bark of Cornus walteri that Control Adipocyte and Osteoblast Differentiations
Source: Molecules. 2018 Oct 23;23(11):2732. doi: 10.3390/molecules23112732 (PMC6278563; doi:10.3390/molecules23112732)

**Supporting Information**

**Tirucallane triterpenoids from the stems and stem bark of *Cornus walteri* that control adipocyte and osteoblast differentiation**

**Seoung Rak Lee ^1^, Eunyong Choi ^2^, Se Hun Jeon ^3^, Xue Yan Zhi ^3^, Jae Sik Yu ^1^, Seon-Hee Kim ^2^, Jeongmi Lee ^1^, Ki-Moon Park ^3^ and Ki Hyun Kim ^1^,***

^1^School of Pharmacy, Sungkyunkwan University, Suwon 16419, Republic of Korea; davidseoungrak@gmail.com (S.R.L.); [jsyu@bu.edu](mailto:jsyu@bu.edu) (J.S.Y.); [jlee0610@skku.edu](mailto:jlee0610@skku.edu) (J.L.). ^2^Sungkyun Biotech Co. LTD., Suwon 16419, Republic of Korea; eychoi8812@sungkyunbiotech.co.kr (E.C.); seonhee31@gmail.com (S.-H.K.). ^3^School of Biotechnology and Bioengineering, Sungkyunkwan University, Suwon 16419, Republic of Korea; shjeon0507@gmail.com (S.H.J.); [zhixueyan0214@gmail.com](mailto:zhixueyan0214@gmail.com) (X.Y.Z.); [pkm1001@skku.edu](mailto:pkm1001@skku.edu) (K.-M.P.)

*** Corresponding author:

Ki Hyun Kim, Tel: +82-31-290-7700; Fax: +82-31-290-7730; E-mail: khkim83@skku.edu

**Supporting Information Contents:**

**Figure S1.** The ^1^H NMR spectrum of **1** (CDCl_3_, 500 MHz)...…..……………………………………………………………………………….4

**Figure S2.** The ^13^C NMR spectrum of **1** (CDCl_3_, 125 MHz)..……………………………………….…………………….……………………5

**Figure S3.** The ^1^H-^1^H COSY spectrum of **1** (CDCl_3_)……………………………………………………………………………………………6

**Figure S4.** The expanded key ^1^H-^1^H COSY spectrum of **1** (CDCl_3_) …………………………………………………………………………7-8

**Figure S5.** The HMQC spectrum of **1** (CDCl_3_) …………………………………………………………………………………………… ……9

**Figure S6.** The expanded key HMQC spectrum of **1** (CDCl_3_) …………………………………………………………………………………10

**Figure S7.** The HMBC spectrum of **1** (CDCl_3_) …………………………………………………………………………………………………11

**Figure S8.** The expanded key HMBC spectrum of **1** (CDCl_3_) …………………………………………………………………………………12

**Figure S9.** The NOESY spectrum of **1** (CDCl_3_) ………………………………………………………………………………………………13

**Figure S10.** The expanded key NOESY spectrum of **1** (CDCl_3_) ………………………………………………………………………………14

**Figure S11.** The ^1^H NMR spectrum of **2** (CDCl_3_, 500 MHz) …………………………………………………………………………………15

**Figure S12.** The ^13^C NMR spectrum of **2** (CDCl_3_, 125 MHz) …………………………………………………………………………………16

**Figure S13.** The ^1^H-^1^H COSY spectrum of **2** (CDCl_3_) …………………………………………………………………………………………17

**Figure S14.** The expanded key ^1^H-^1^H COSY spectrum of **2** (CDCl_3_) …………………………………………………………………………18

**Figure S15.** The HSQC spectrum of **2** (CDCl_3_) ………………………………………………………………………………………………19

**Figure S16.** The expanded key HSQC spectrum of **2** (CDCl_3_) …………………………………………………………………………………20

**Figure S17.** The HMBC spectrum of **2** (CDCl_3_) ………………………………………………………………………………………………21

**Figure S18.** The expanded key HMBC spectrum of **2** (CDCl_3_) ………………………………………………………………………………22

**Figure S19.** The NOESY spectrum of **2** (CDCl_3_) ………………………………………………………………………………………………23

**Figure S20.** The expanded key NOESY spectrum of **2** (CDCl_3_) ………………………………………………………………………………24

**Figure S21.** The ^1^H NMR spectrum of **3** (CDCl_3_, 500 MHz) …………………………………………………………………………………25

**Figure S22.** The ^13^C NMR spectrum of **3** (CDCl_3_, 125 MHz) …………………………………………………………………………………26

**Figure S23.** The ^1^H-^1^H COSY spectrum of **3** (CDCl_3_) …………………………………………………………………………………………27

**Figure S24.** The expanded key ^1^H-^1^H COSY spectrum of **3** (CDCl_3_) …………………………………………………………………………28

**Figure S25.** The HSQC spectrum of **3** (CDCl_3_) ………………………………………………………………………………………………29

**Figure S26.** The expanded key HSQC spectrum of **3** (CDCl_3_) …………………………………………………………………………………30

**Figure S27.** The HMBC spectrum of **3** (CDCl_3_) ………………………………………………………………………………………………31

**Figure S28.** The expanded key HMBC spectrum of **3** (CDCl_3_) ………………………………………………………………………………32

**Figure S29.** The NOESY spectrum of **3** (CDCl_3_) ………………………………………………………………………………………………33

**Figure S30.** The expanded key NOESY spectrum of **3** (CDCl_3_) ………………………………………………………………………………34

**Figure S31.** DP4+ analysis of compound **1** with **1a**, **1b**, **1c**, and **1d** corresponding to isomers 1, 2, 3, and 4….…………………35

**Figure S32.** DP4+ analysis of compound **2** with **2a** and **2b** corresponding to isomers 1 and 2………………….…………………36

**Figure S33.** DP4+ analysis of compound **3** with **3a** and **3b** corresponding to isomers 1 and 2……………….……………………37

**Figure S34.** Optimized ground state structure and NMR shielding constants for chemical shift calculation of isomer 1 of compound **1**……38

**Figure S35.** Optimized ground state structure and NMR shielding constants for chemical shift calculation of isomer 2 of compound **1**……39

**Figure S36.** Optimized ground state structure and NMR shielding constants for chemical shift calculation of isomer 3 of compound **1**……40

**Figure S37.** Optimized ground state structure and NMR shielding constants for chemical shift calculation of isomer 4 of compound **1**……41

**Figure S38.** Optimized ground state structure and NMR shielding constants for chemical shift calculation of isomer 1 of compound **2**……42

**Figure S39.** Optimized ground state structure and NMR shielding constants for chemical shift calculation of isomer 2 of compound **2**……43

**Figure S40.** Optimized ground state structure and NMR shielding constants for chemical shift calculation of isomer 1 of compound **3**……44

**Figure S41.** Optimized ground state structure and NMR shielding constants for chemical shift calculation of isomer 2 of compound **3**……45

**Figure S1.** The ^1^H NMR spectrum of **1** (CDCl_3_, 500 MHz)


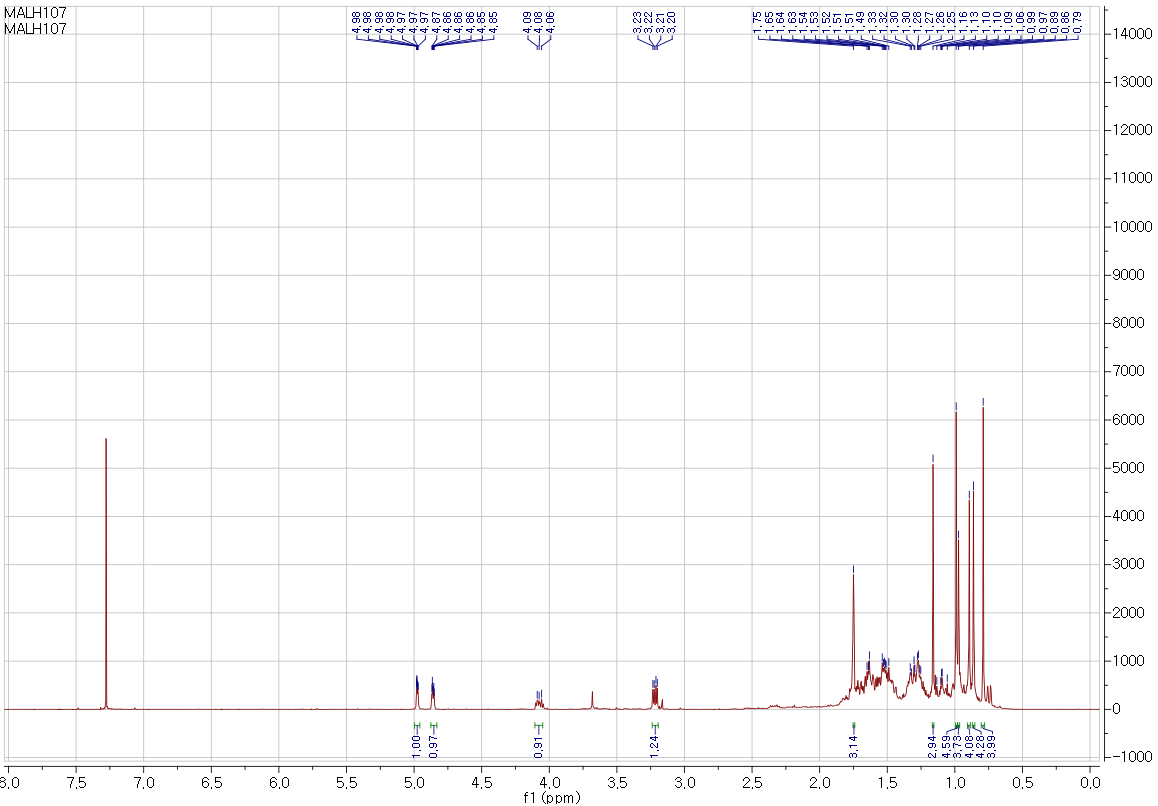


**Figure S2.** The ^13^C NMR spectrum of **1** (CDCl_3_, 125 MHz)


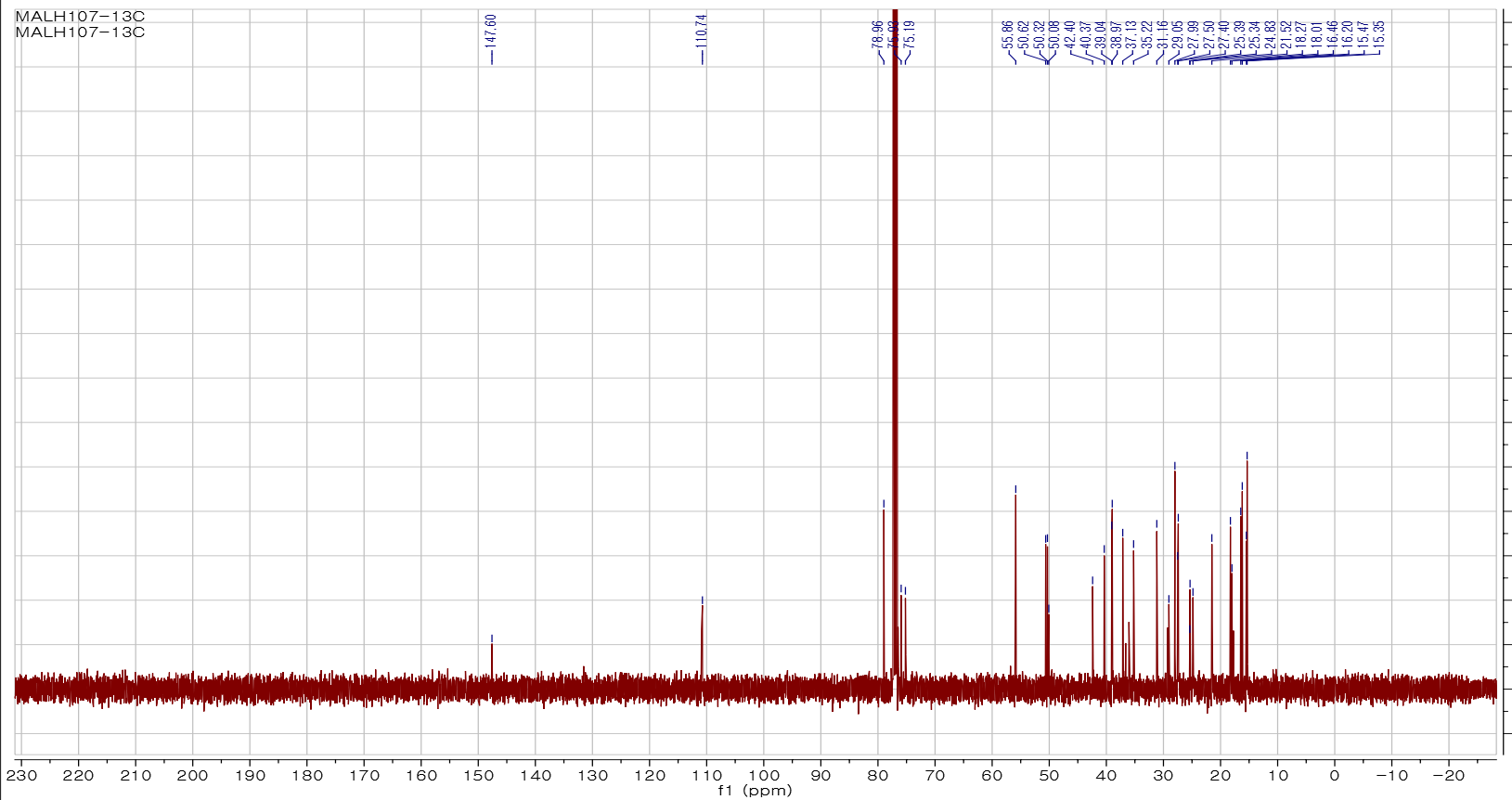


**Figure S3.** The ^1^H-^1^H COSY spectrum of **1** (CDCl_3_)


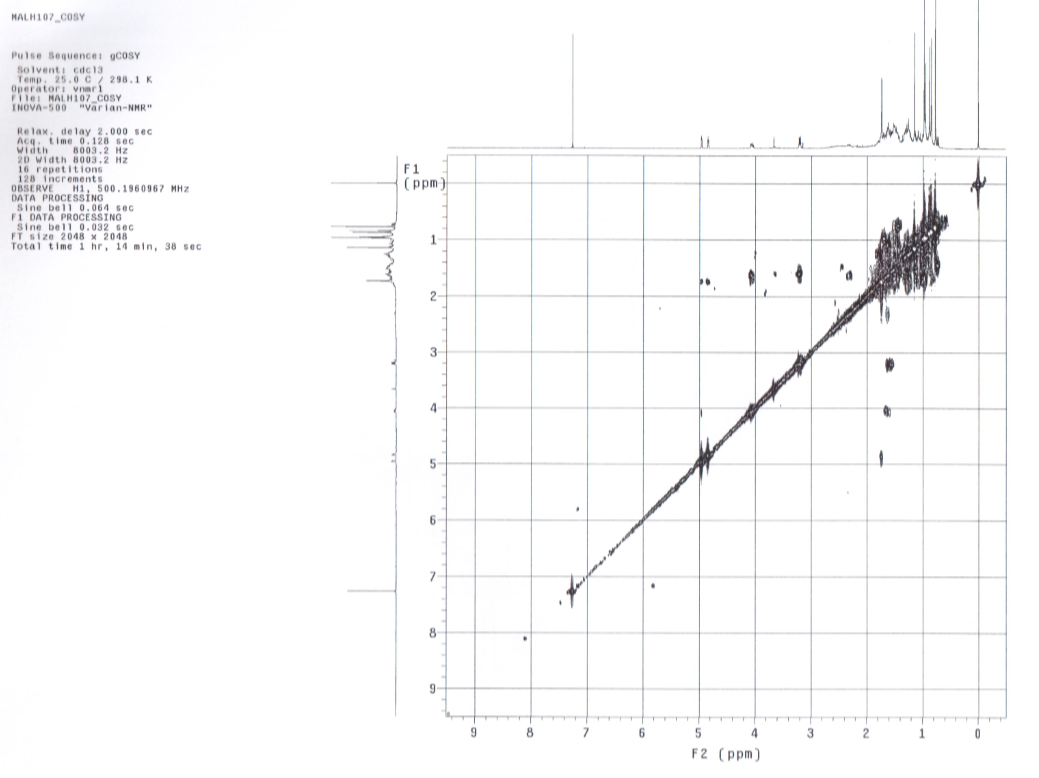


**Figure S4.** The expanded key ^1^H-^1^H COSY spectrum of **1** (CDCl_3_)


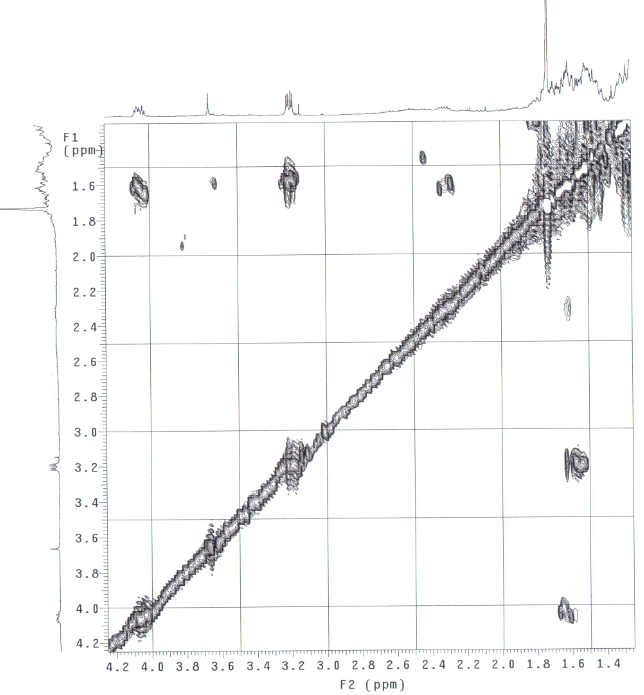


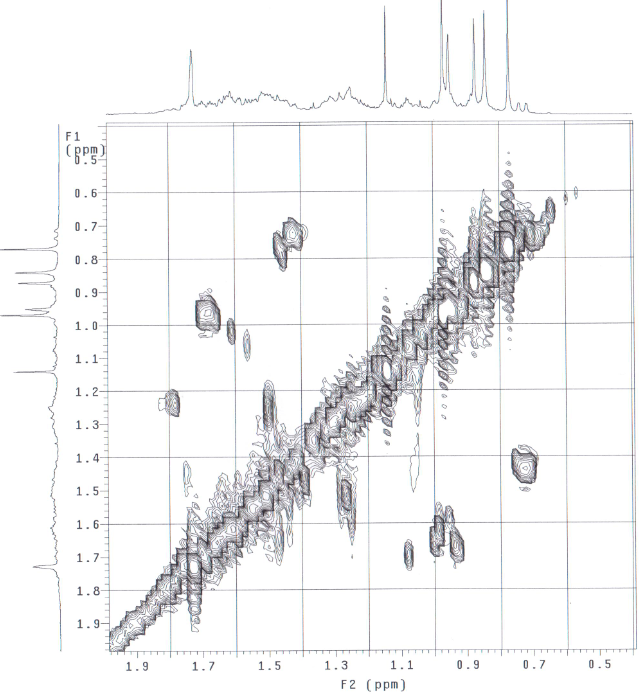


**Figure S5.** The HMQC spectrum of **1** (CDCl_3_)


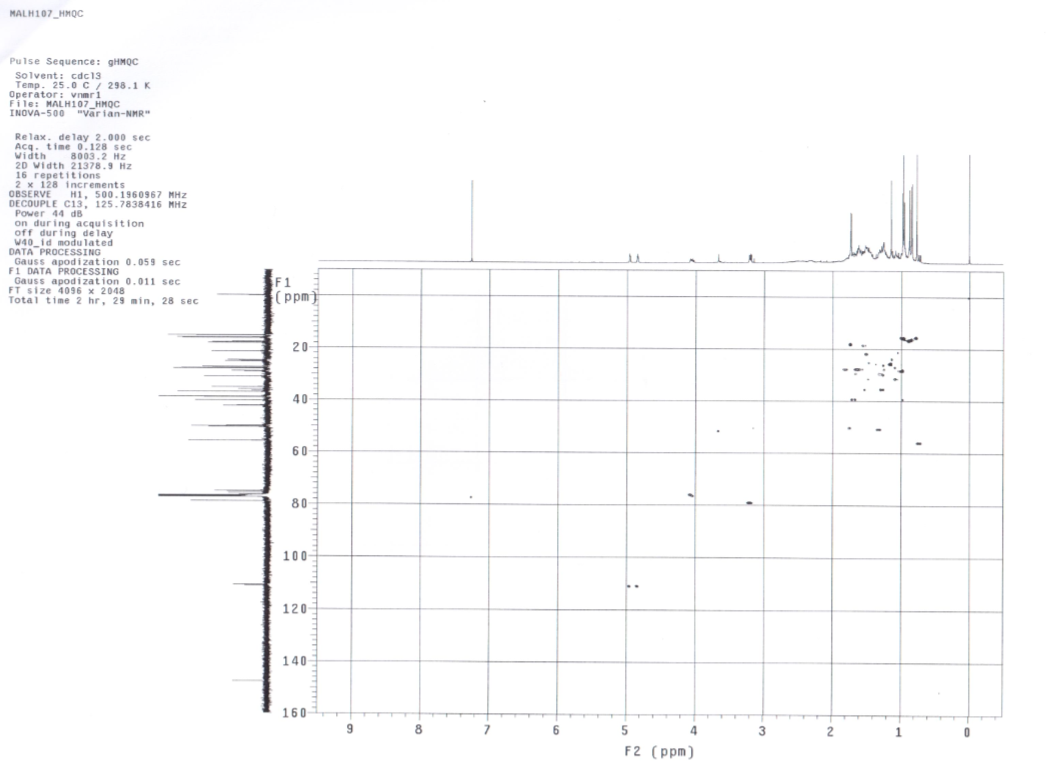


**Figure S6.** The expanded key HMQC spectrum of **1** (CDCl_3_)


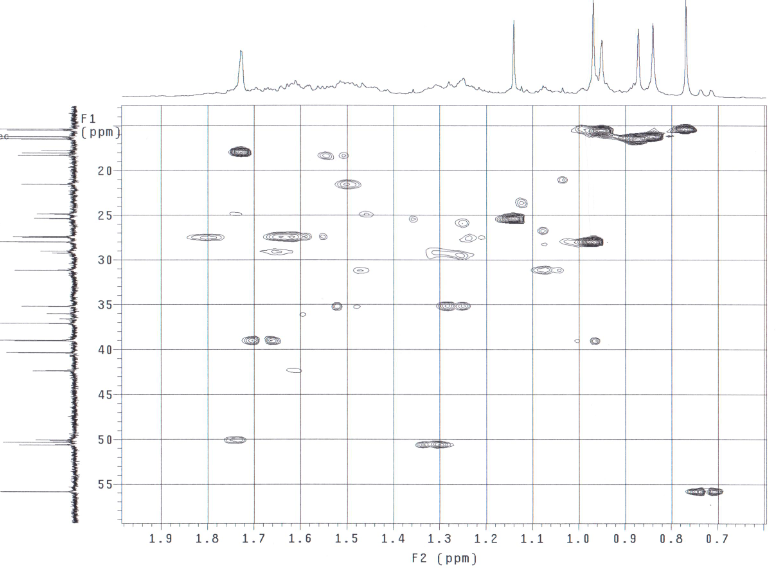


**Figure S7.** The HMBC spectrum of **1** (CDCl_3_)


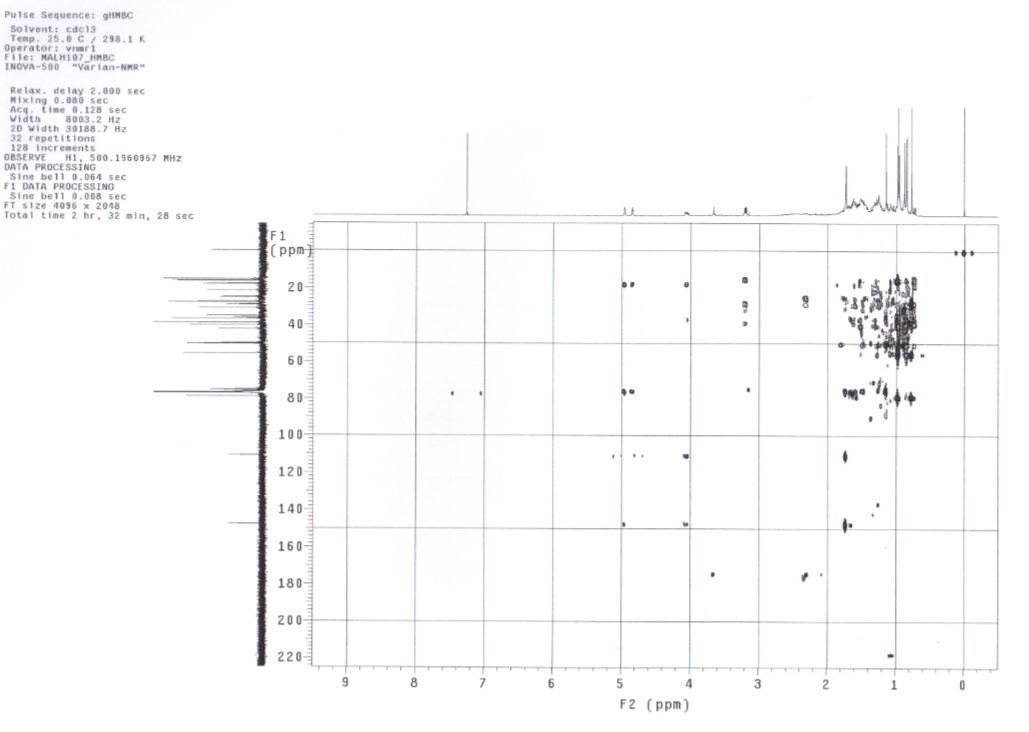


**Figure S8.** The expanded key HMBC spectrum of **1** (CDCl_3_)


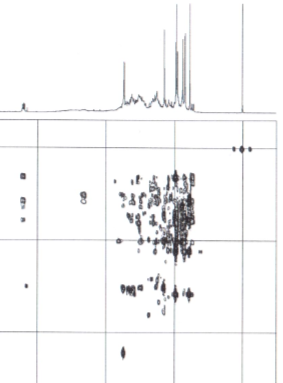


**Figure S9.** The NOESY spectrum of **1** (CDCl_3_)


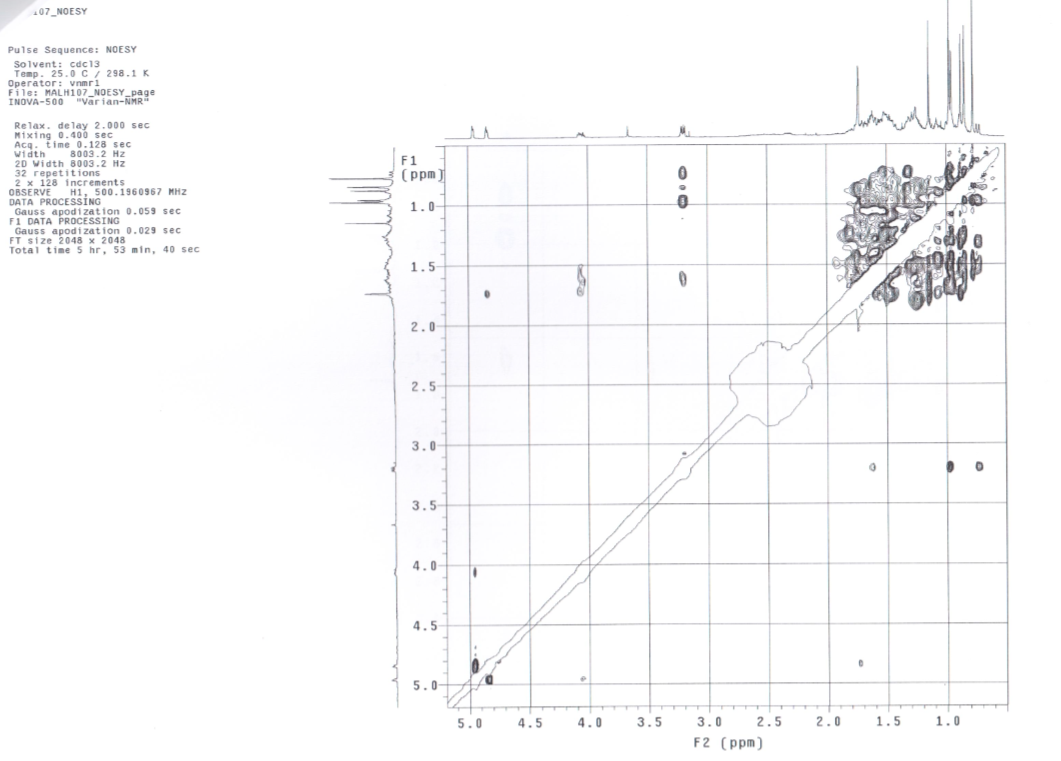


**Figure S10.** The expanded key NOESY spectrum of **1** (CDCl_3_)


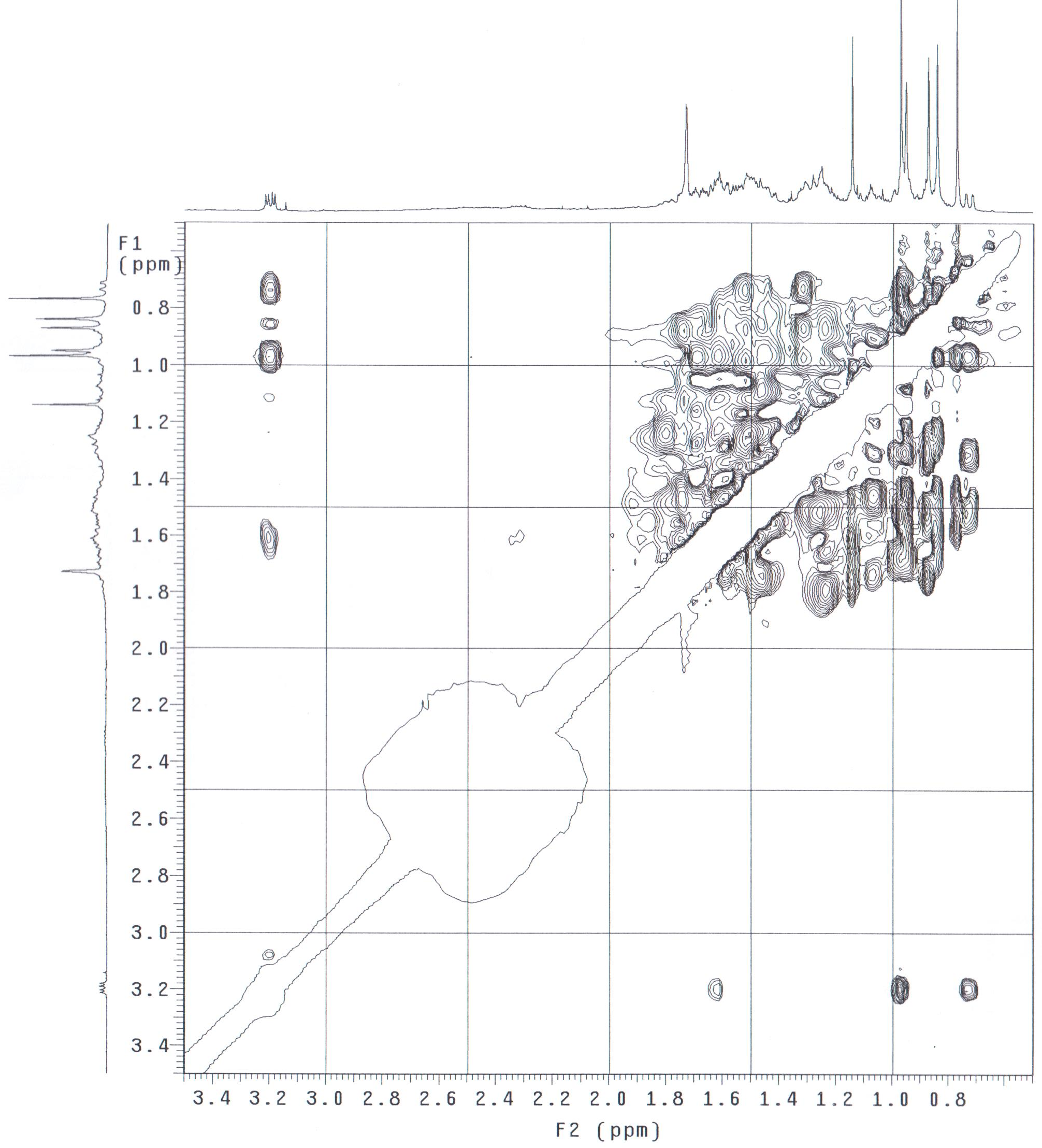


**Figure S11.** The ^1^H NMR spectrum of **2** (CDCl_3_, 500 MHz)


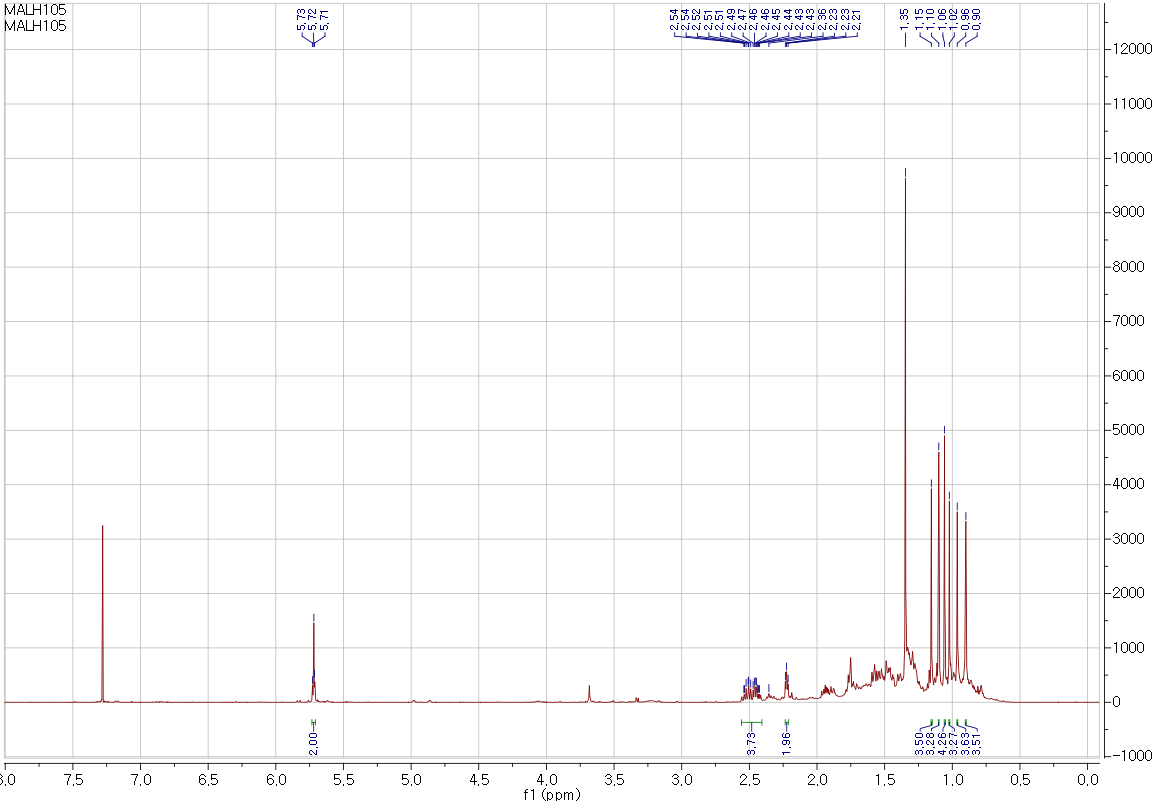


**Figure S12.** The ^13^C NMR spectrum of **2** (CDCl_3_, 125 MHz)


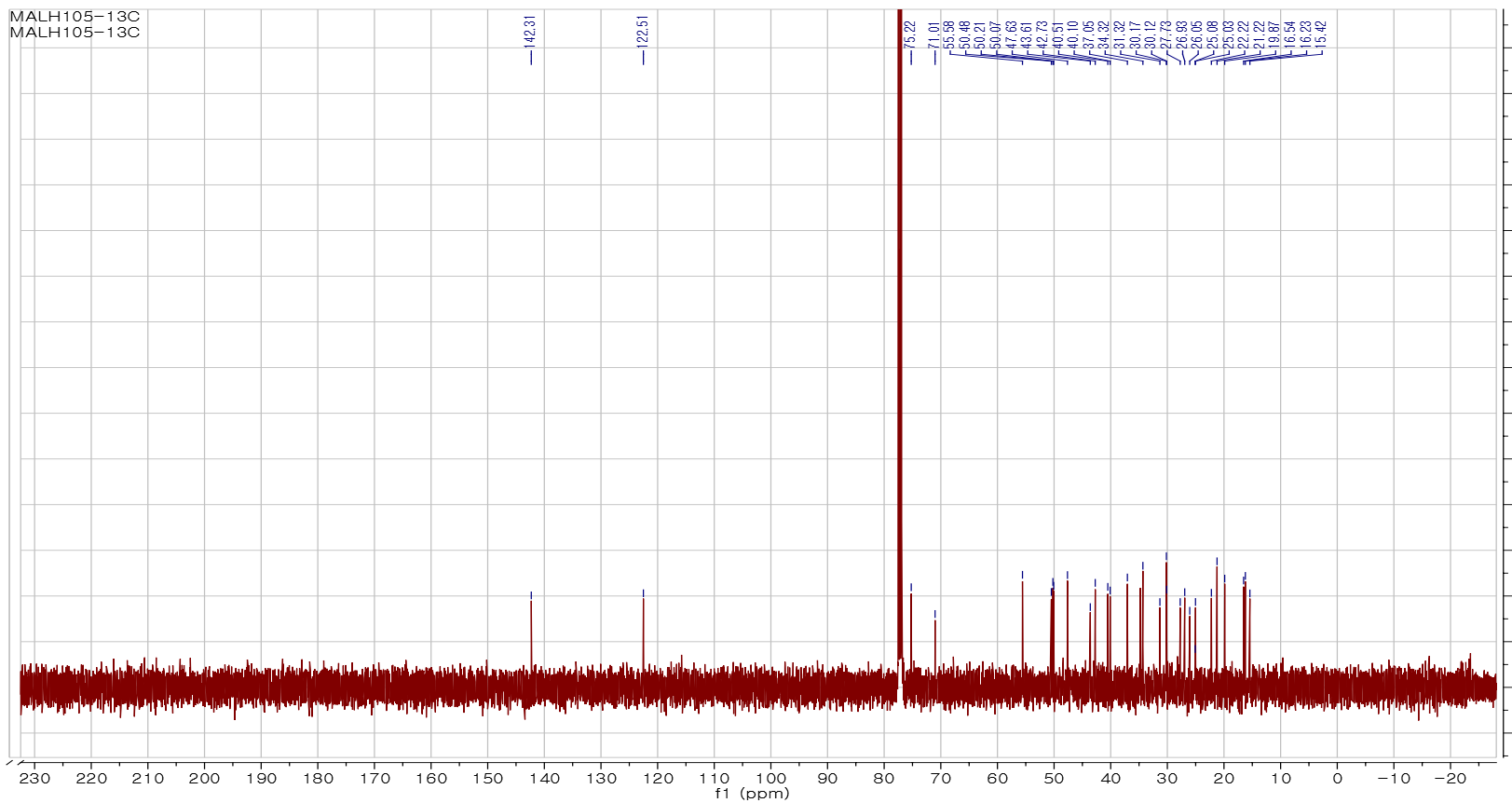


**Figure S13.** The ^1^H-^1^H COSY spectrum of **2** (CDCl_3_)

**Figure S14.** The expanded key ^1^H-^1^H COSY spectrum of **2** (CDCl_3_)


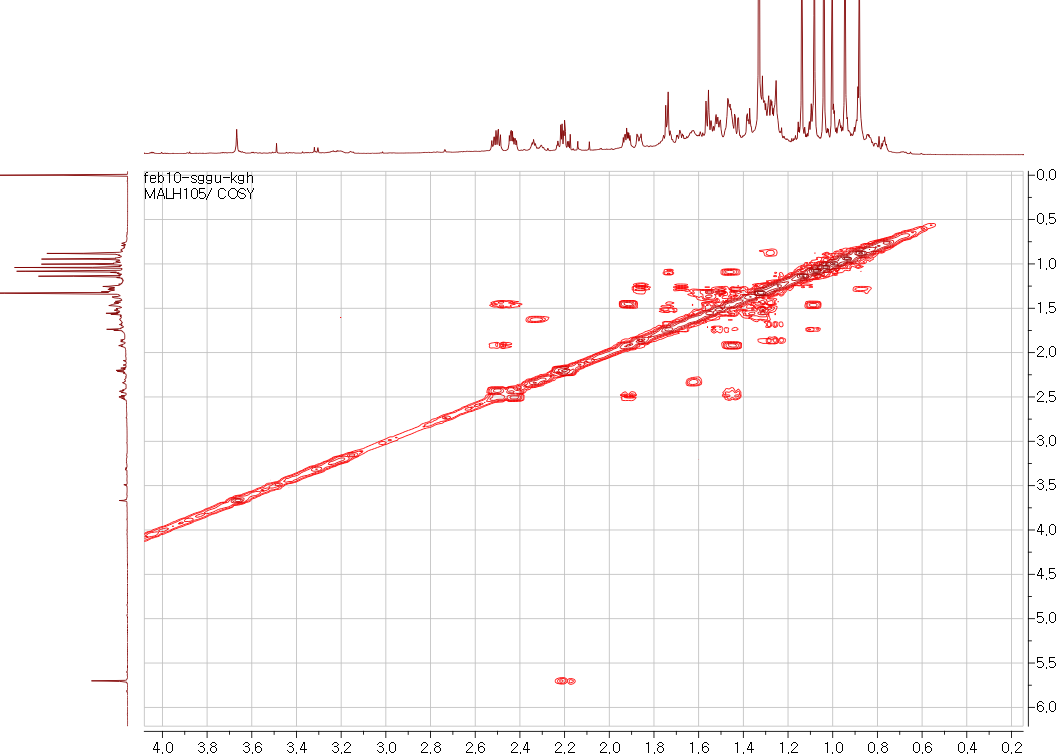


**Figure S15.** The HSQC spectrum of **2** (CDCl_3_)


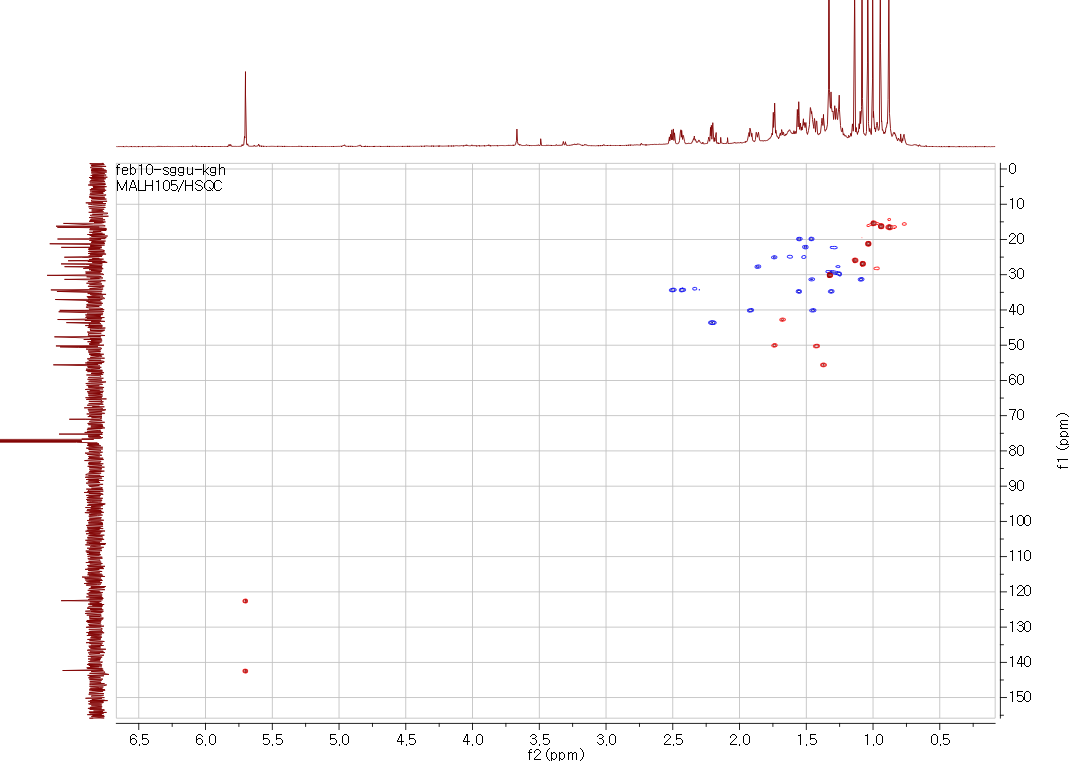


**Figure S16.** The expanded key HSQC spectrum of **2** (CDCl_3_)


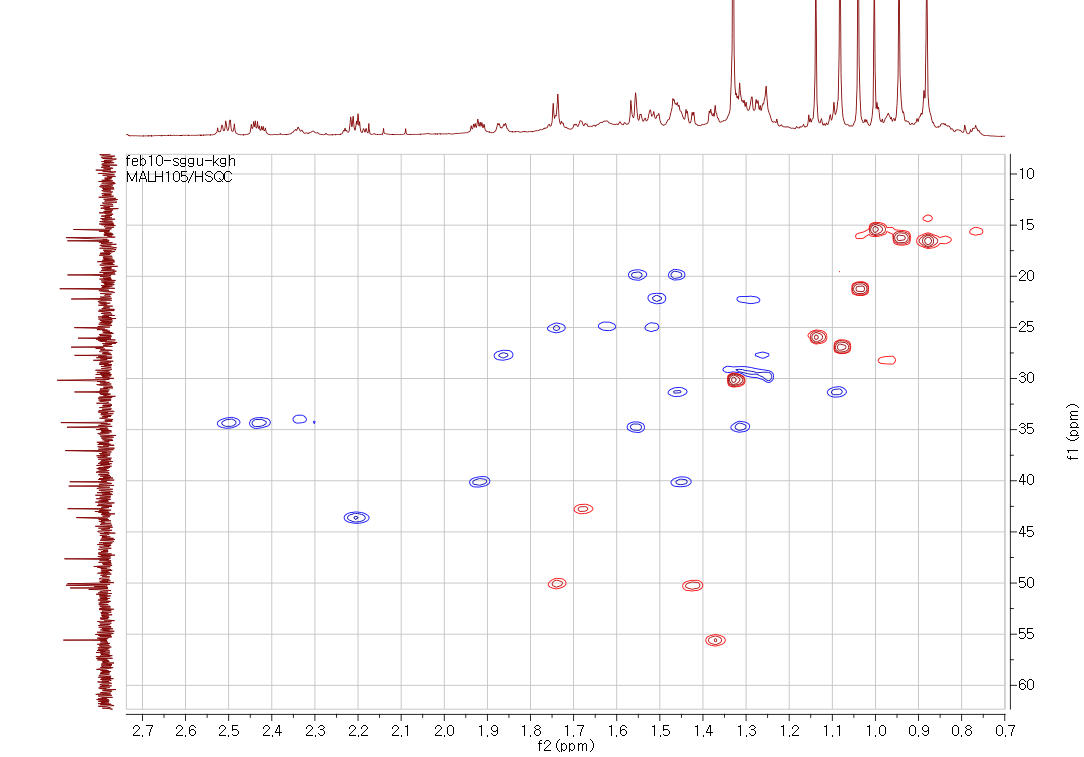


**Figure S17.** The HMBC spectrum of **2** (CDCl_3_)

**Figure S18.** The expanded key HMBC spectrum of **2** (CDCl_3_)


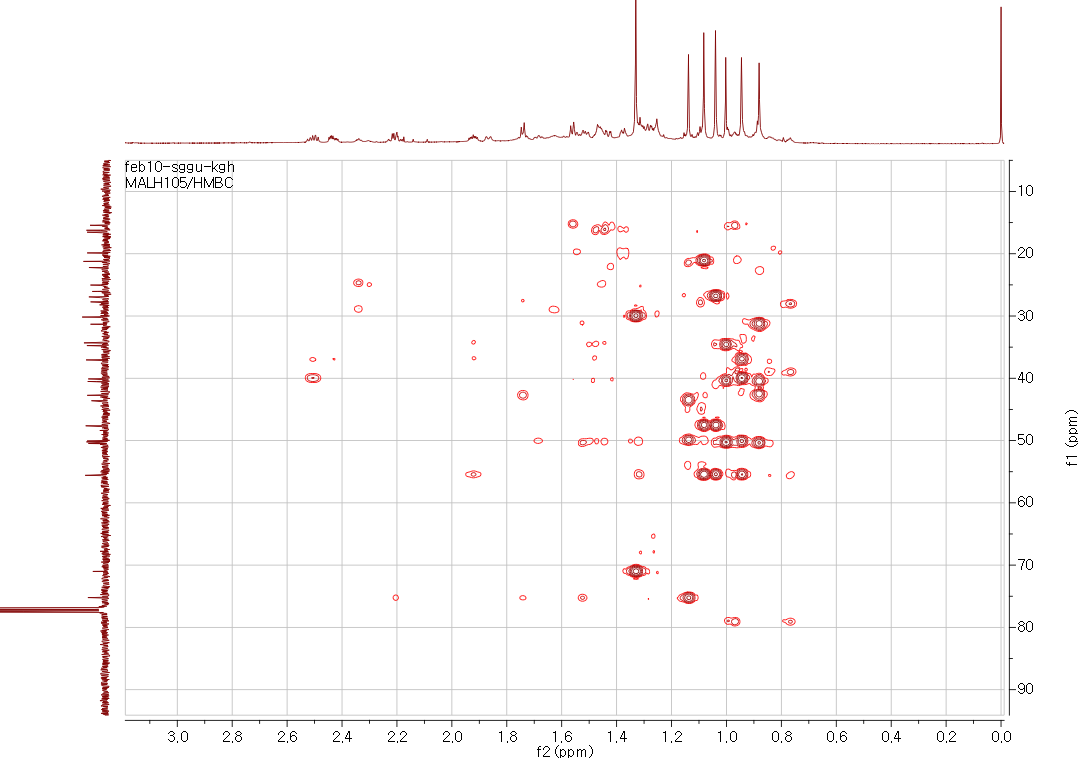


**Figure S19.** The NOESY spectrum of **2** (CDCl_3_)

**Figure S20.** The expanded key NOESY spectrum of **2** (CDCl_3_)


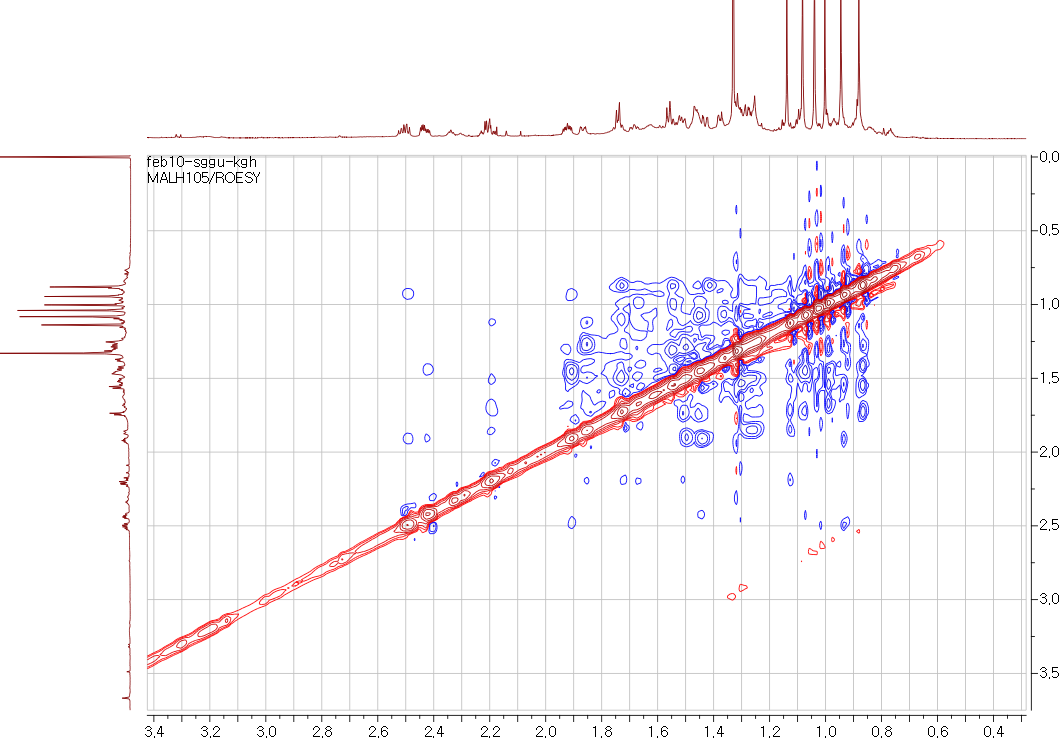


**Figure S21.** The ^1^H NMR spectrum of **3** (CDCl_3_, 500 MHz)


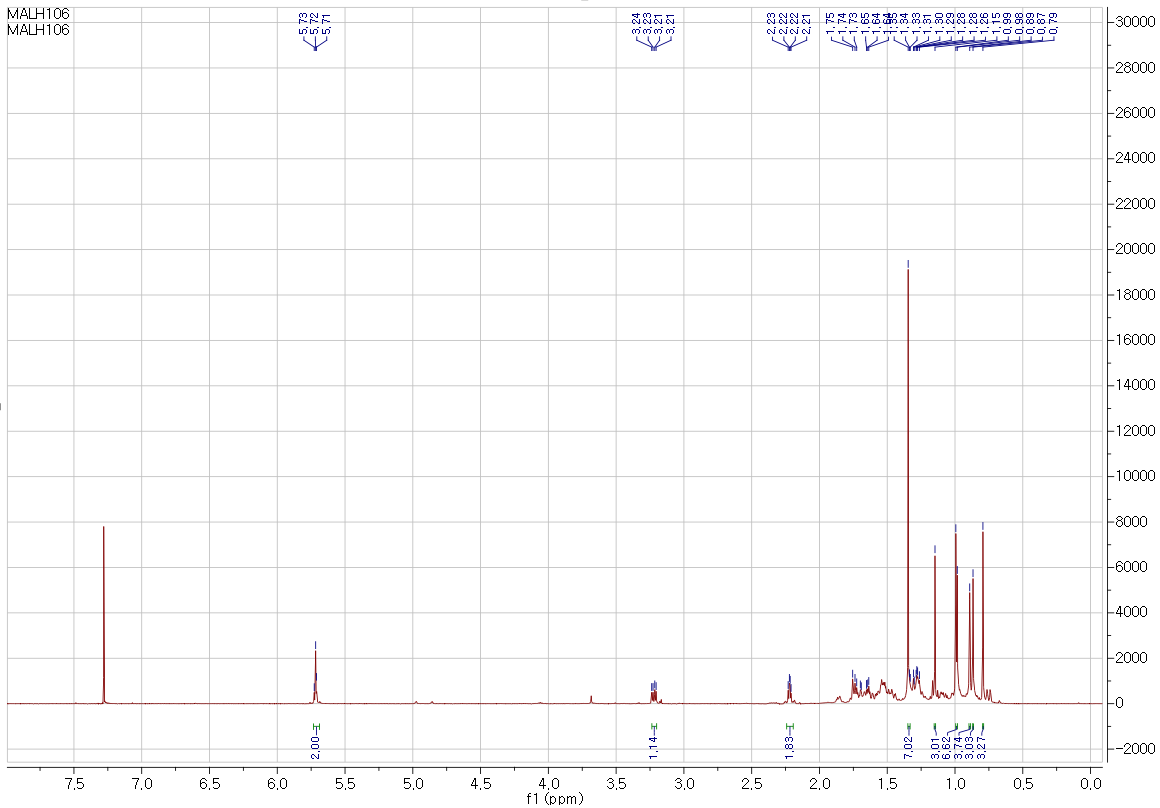


**Figure S22.** The ^13^C NMR spectrum of **3** (CDCl_3_, 125 MHz)


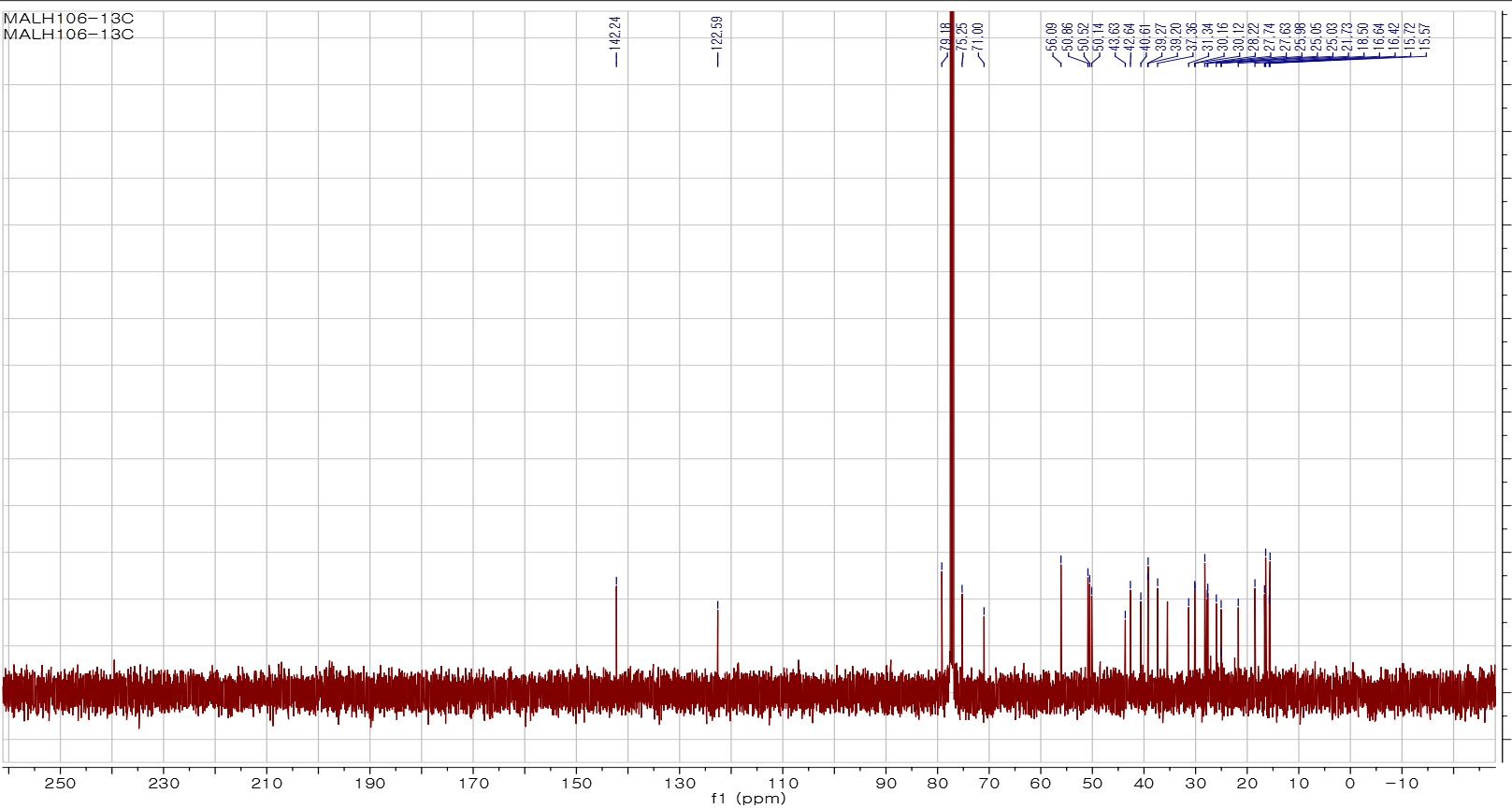


**Figure S23.** The ^1^H-^1^H COSY spectrum of **3** (CDCl_3_)

**Figure S24.** The expanded key ^1^H-^1^H COSY spectrum of **3** (CDCl_3_)


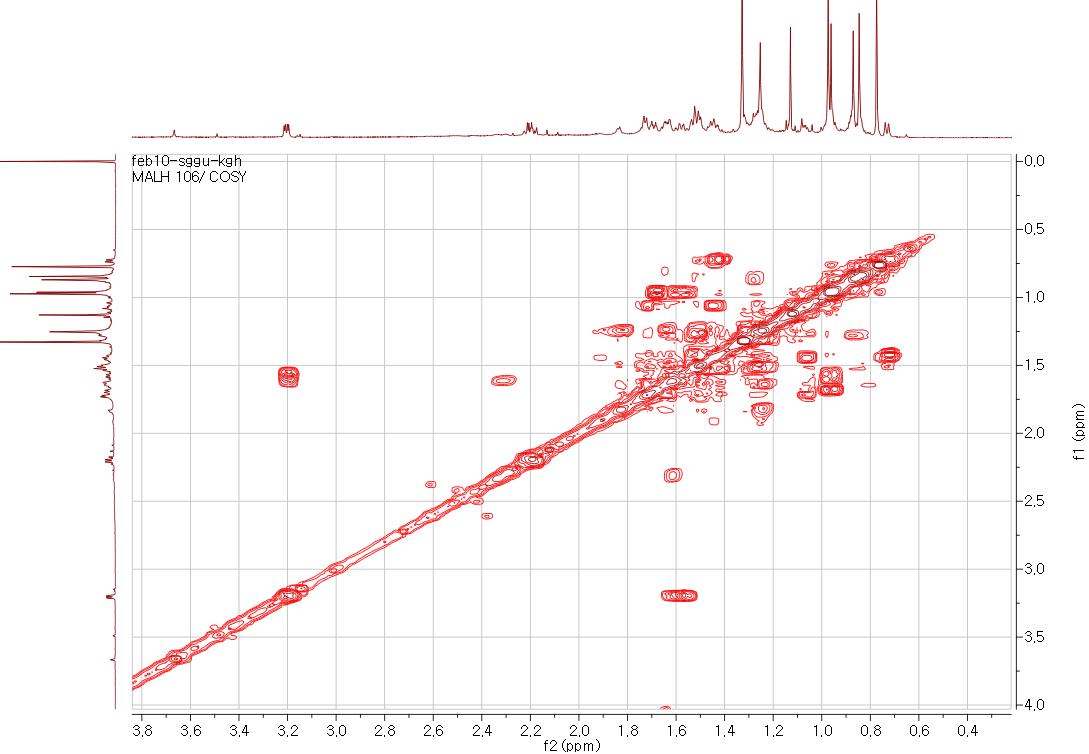


**Figure S25.** The HSQC spectrum of **3** (CDCl_3_)


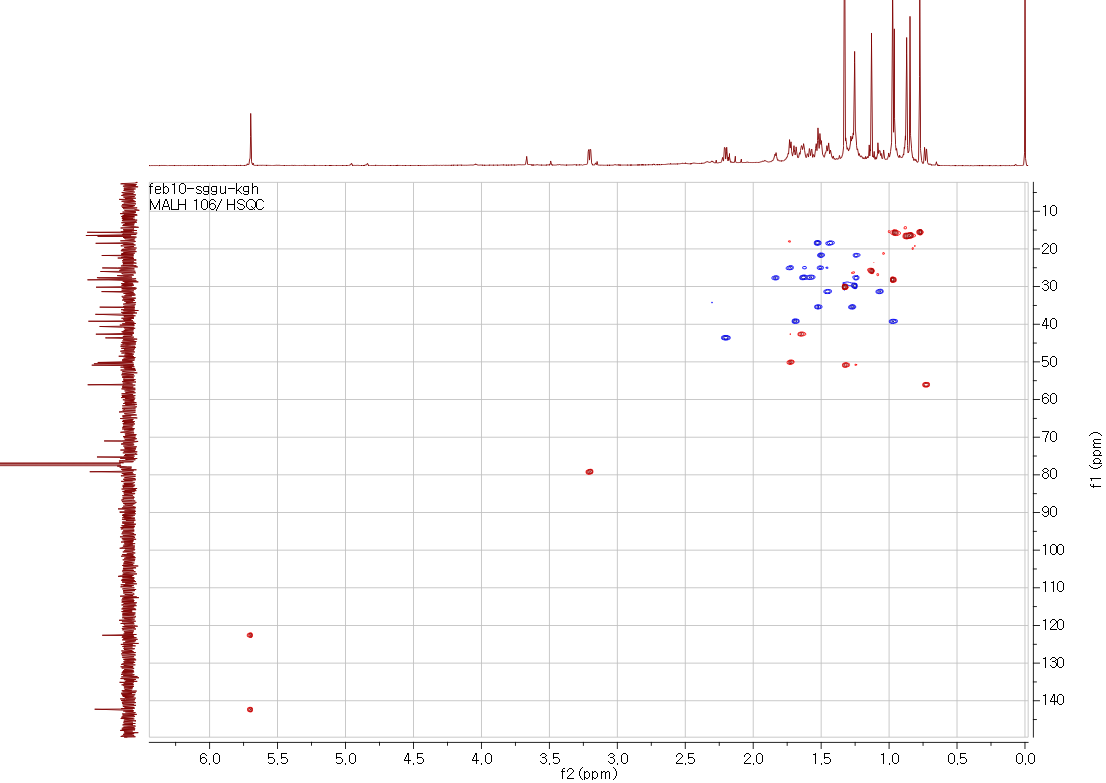


**Figure S26.** The expanded key HSQC spectrum of **3** (CDCl_3_)


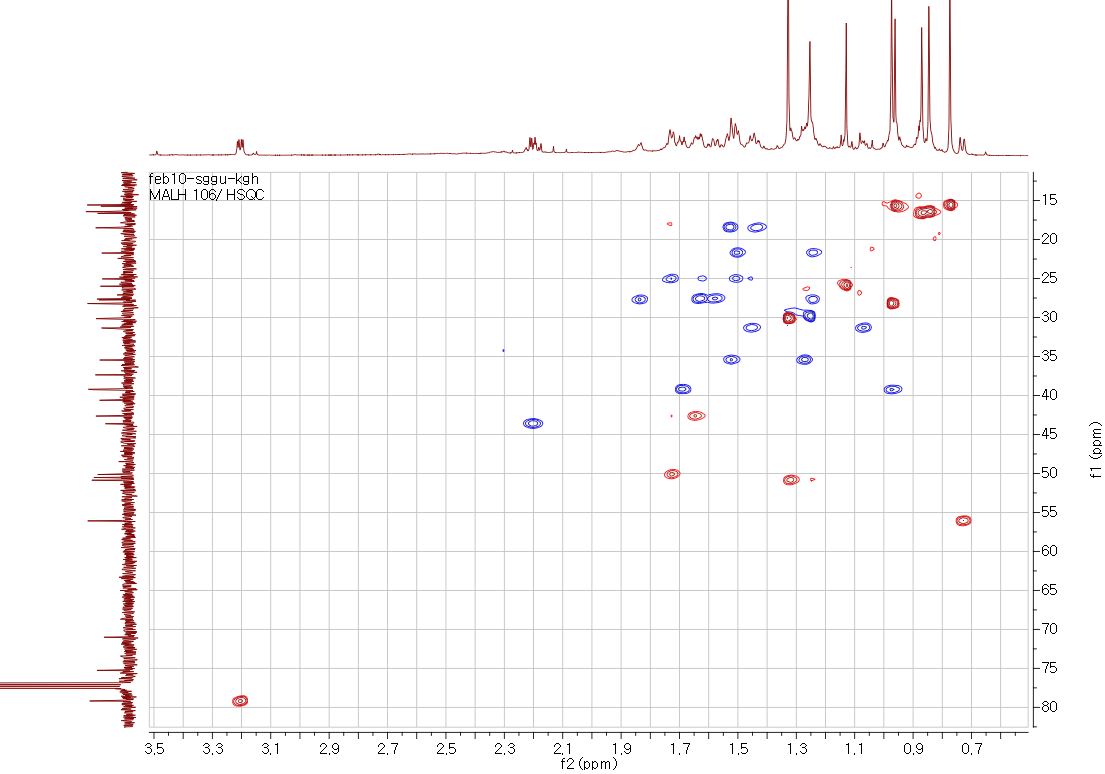


**Figure S27.** The HMBC spectrum of **3** (CDCl_3_)

**Figure S28.** The expanded key HMBC spectrum of **3** (CDCl_3_)


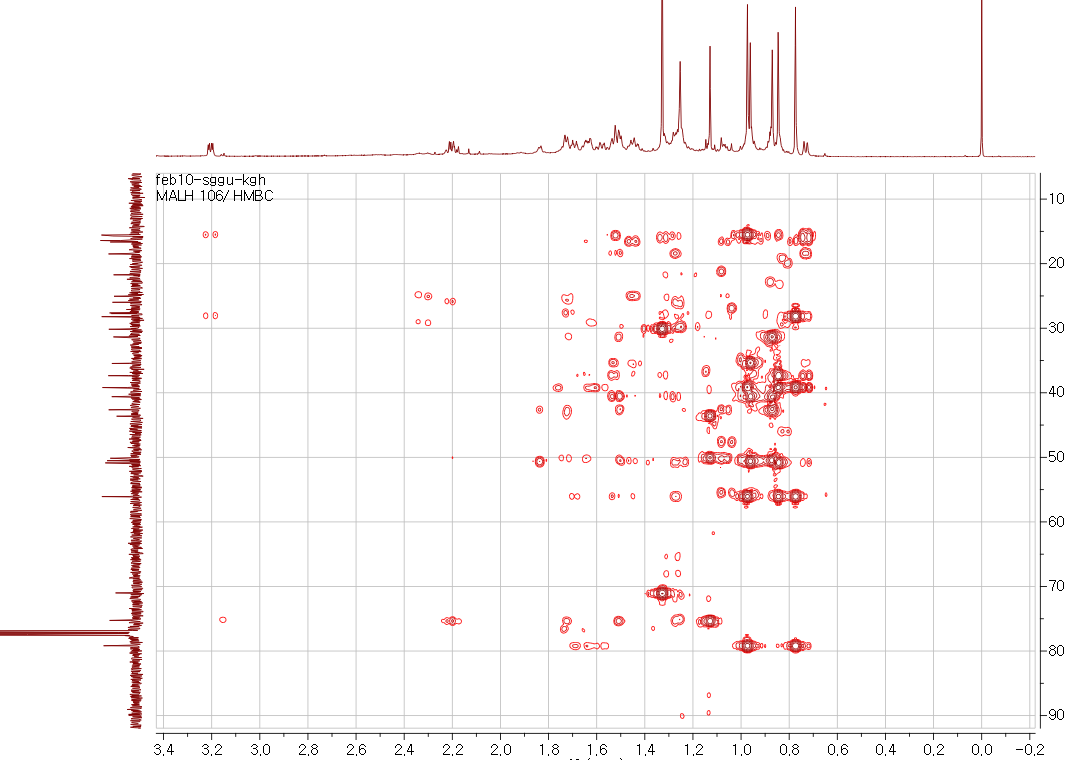


**Figure S29.** The NOESY spectrum of **3** (CDCl_3_)

**Figure S30.** The expanded key NOESY spectrum of **3** (CDCl_3_)


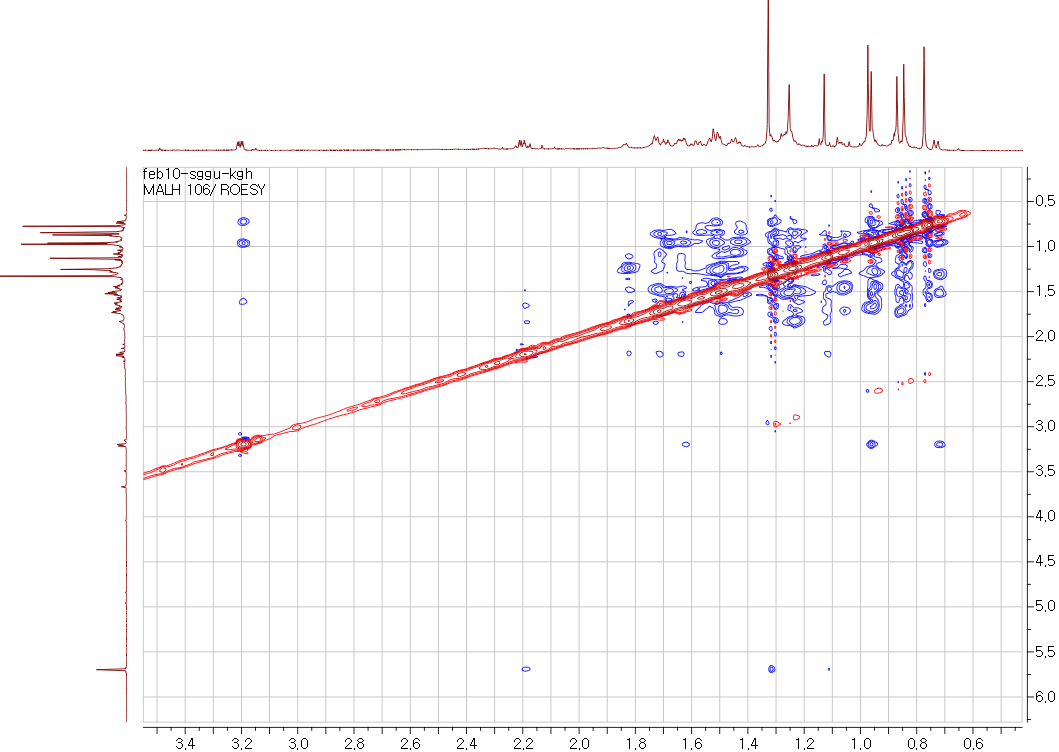


**Figure S31.** DP4+ analysis of compound **1** with **1a**, **1b**, **1c**, and **1d** corresponding to isomers 1, 2, 3, and 4


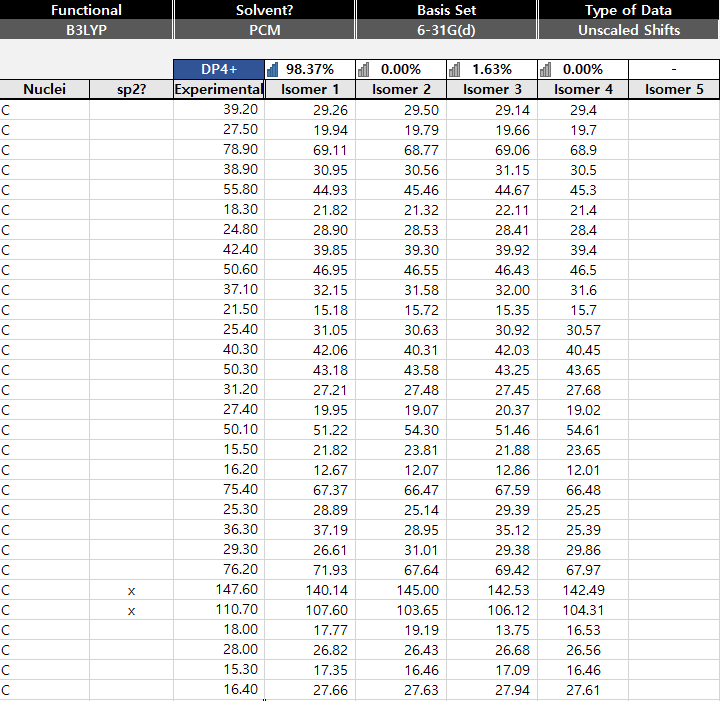


**Figure S32.** DP4+ analysis of compound **2** with **2a** and **2b** corresponding to isomers 1 and 2


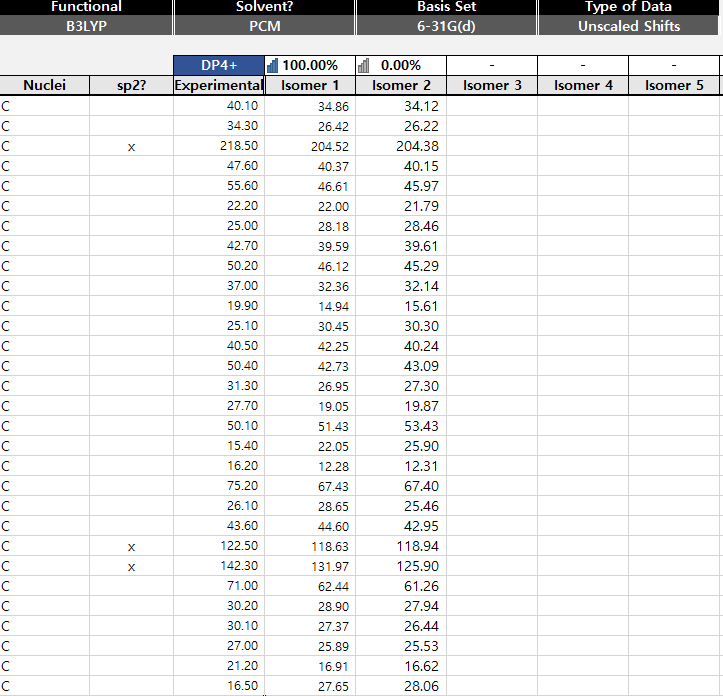


**Figure S33.** DP4+ analysis of compound **3** with **3a** and **3b** corresponding to isomers 1 and 2


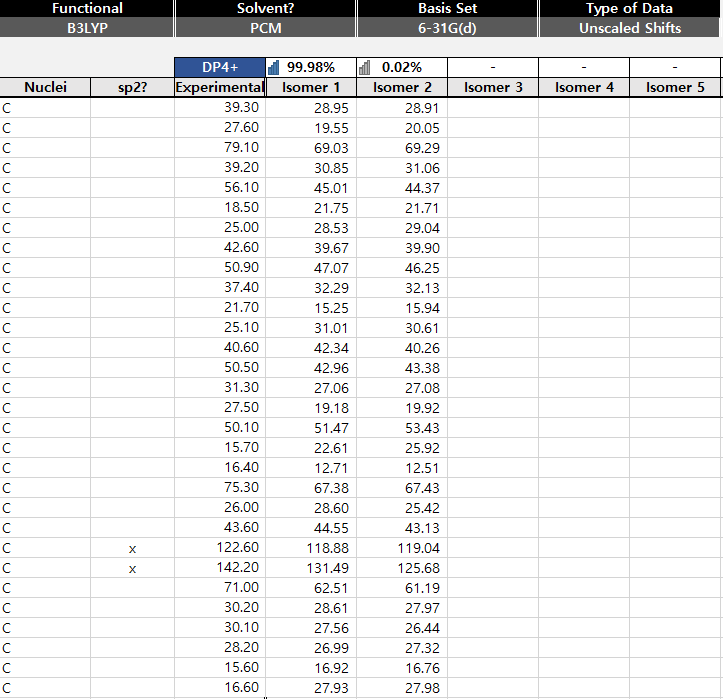


**Figure S34.** Optimized ground state structure and NMR shielding constants for chemical shift calculation of isomer 1 of compound **1**.


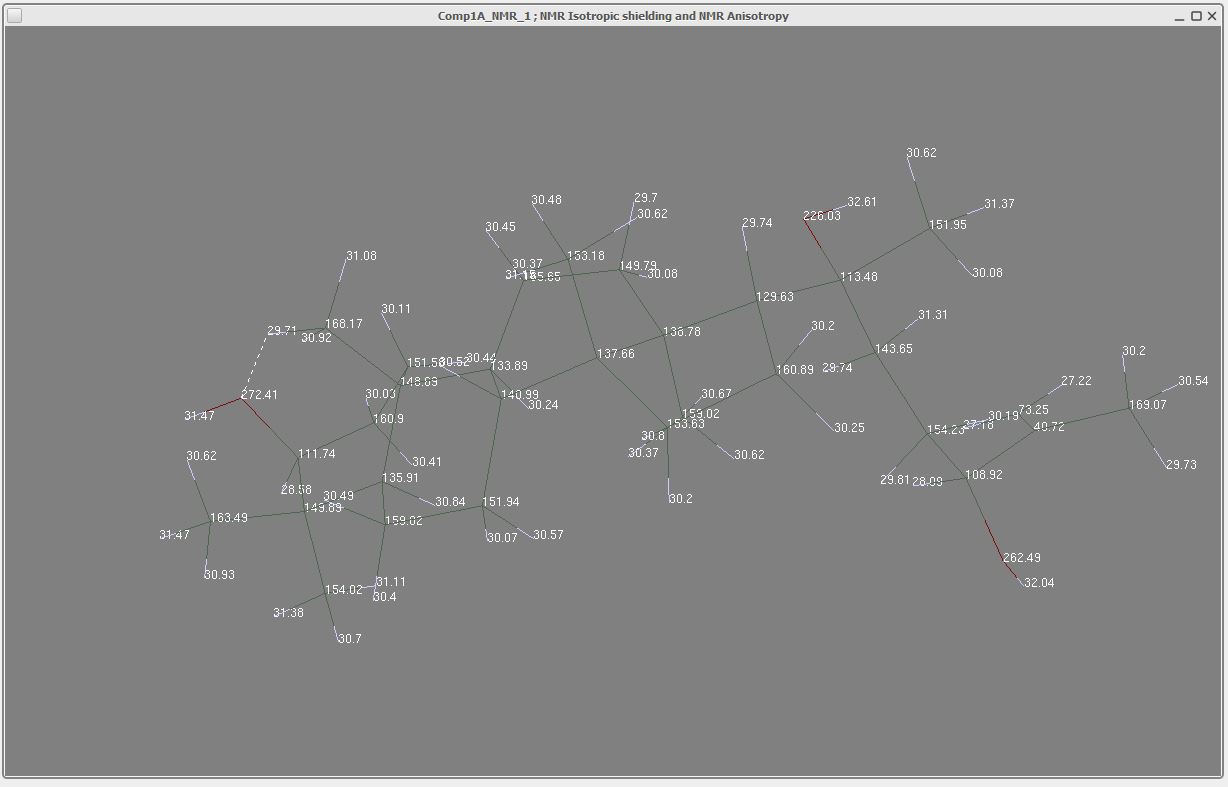


**Figure S35.** Optimized ground state structure and NMR shielding constants for chemical shift calculation of isomer 2 of compound **1**.


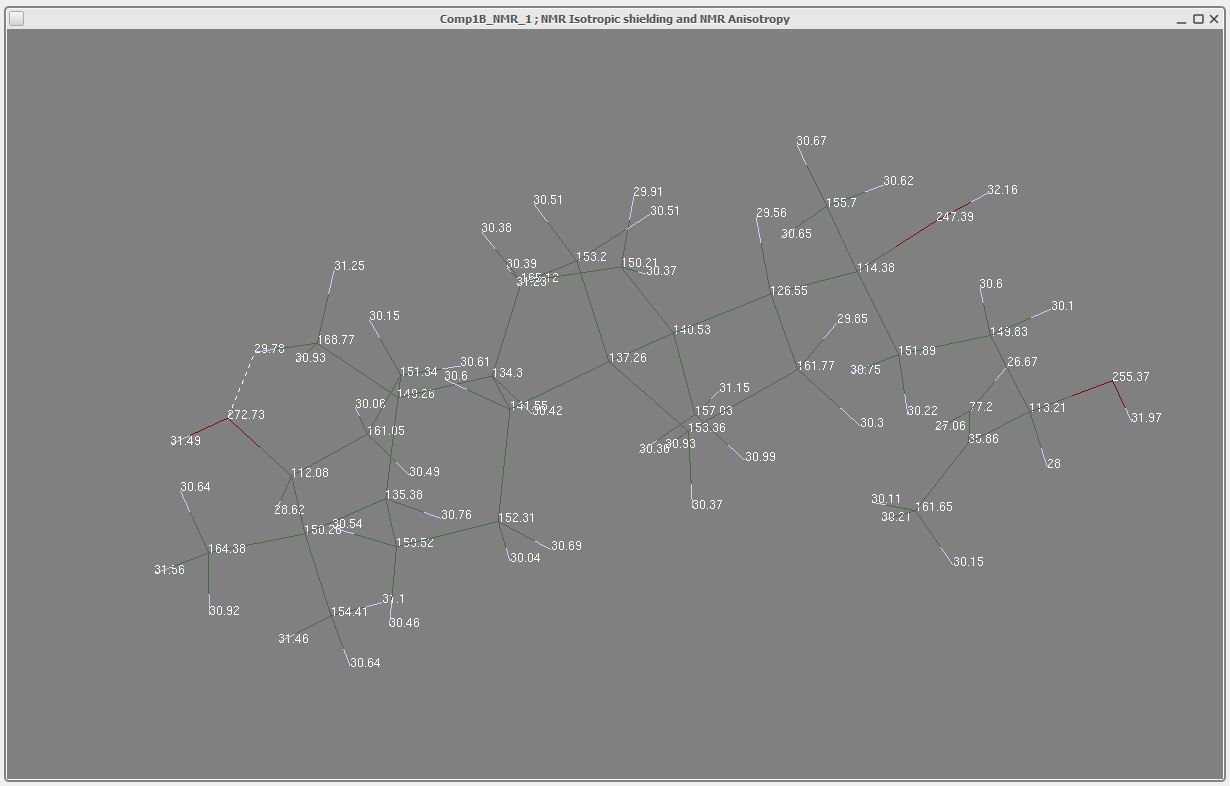


**Figure S36.** Optimized ground state structure and NMR shielding constants for chemical shift calculation of isomer 3 of compound **1**.


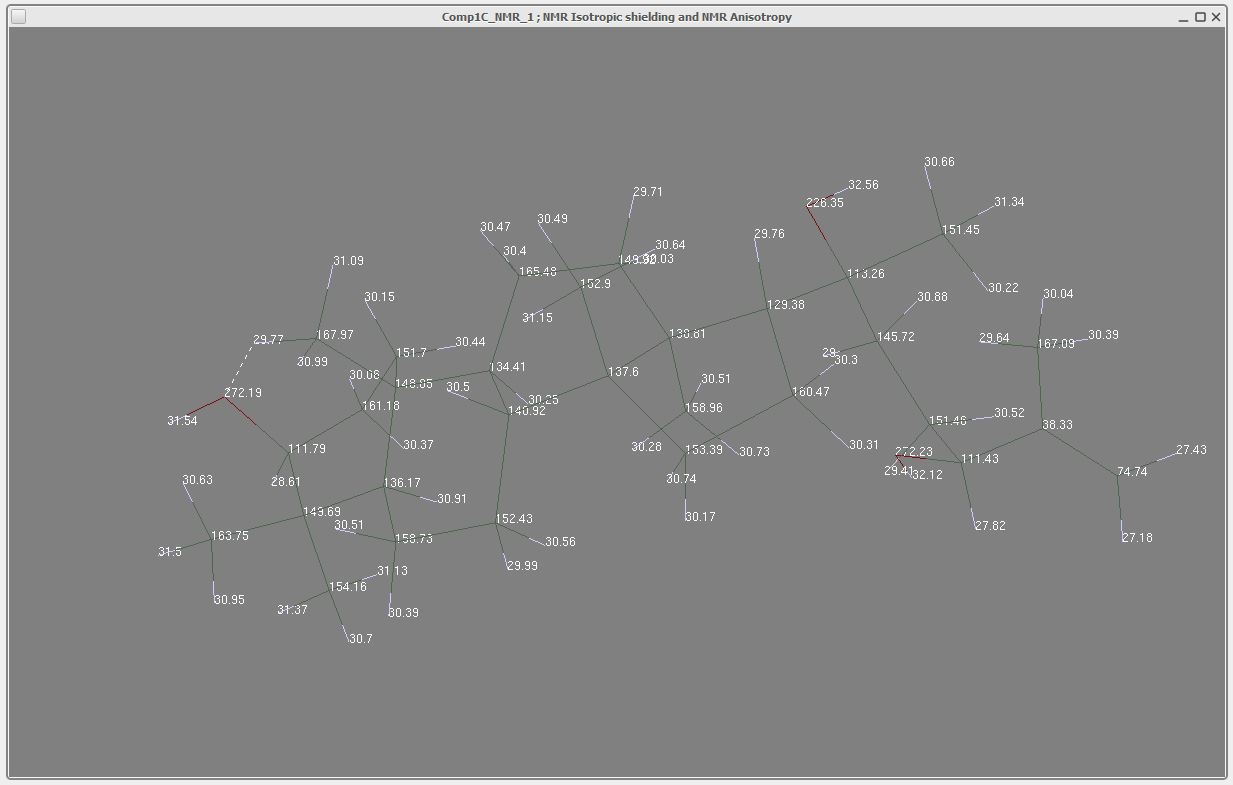


**Figure S37.** Optimized ground state structure and NMR shielding constants for chemical shift calculation of isomer 4 of compound **1**.


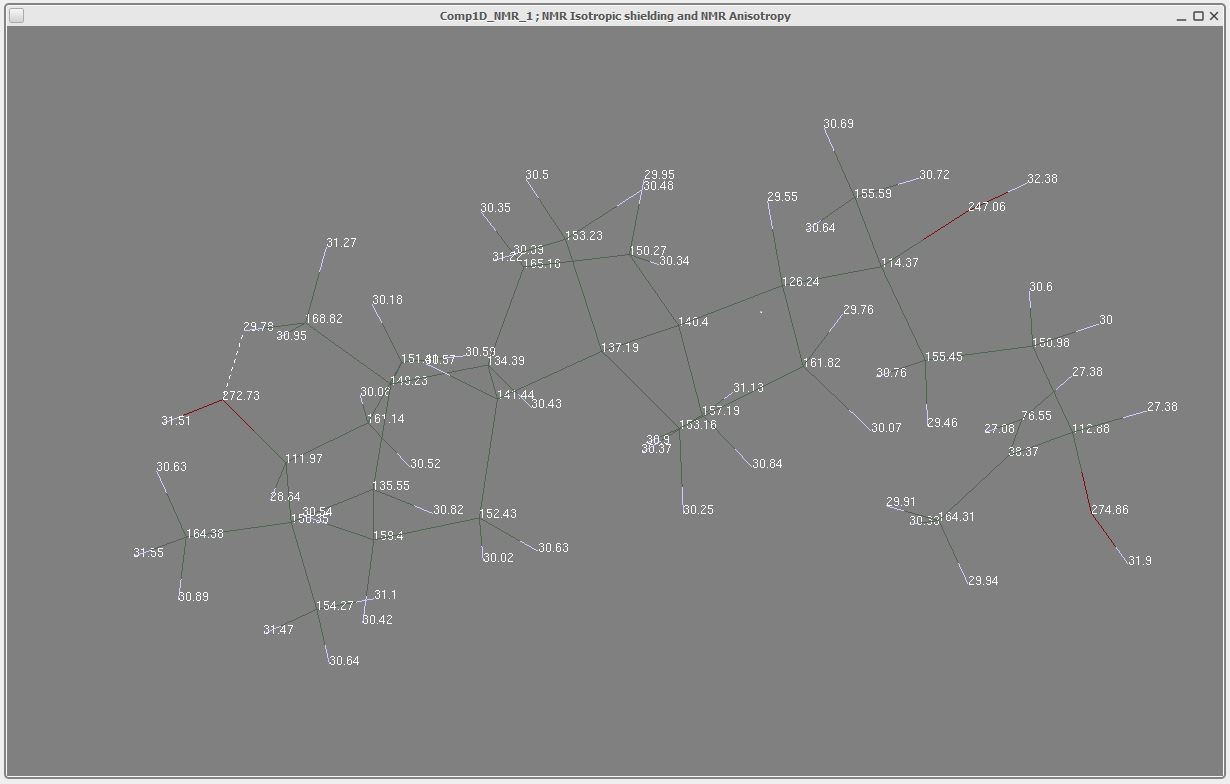


**Figure S38.** Optimized ground state structure and NMR shielding constants for chemical shift calculation of isomer 1 of compound **2**.


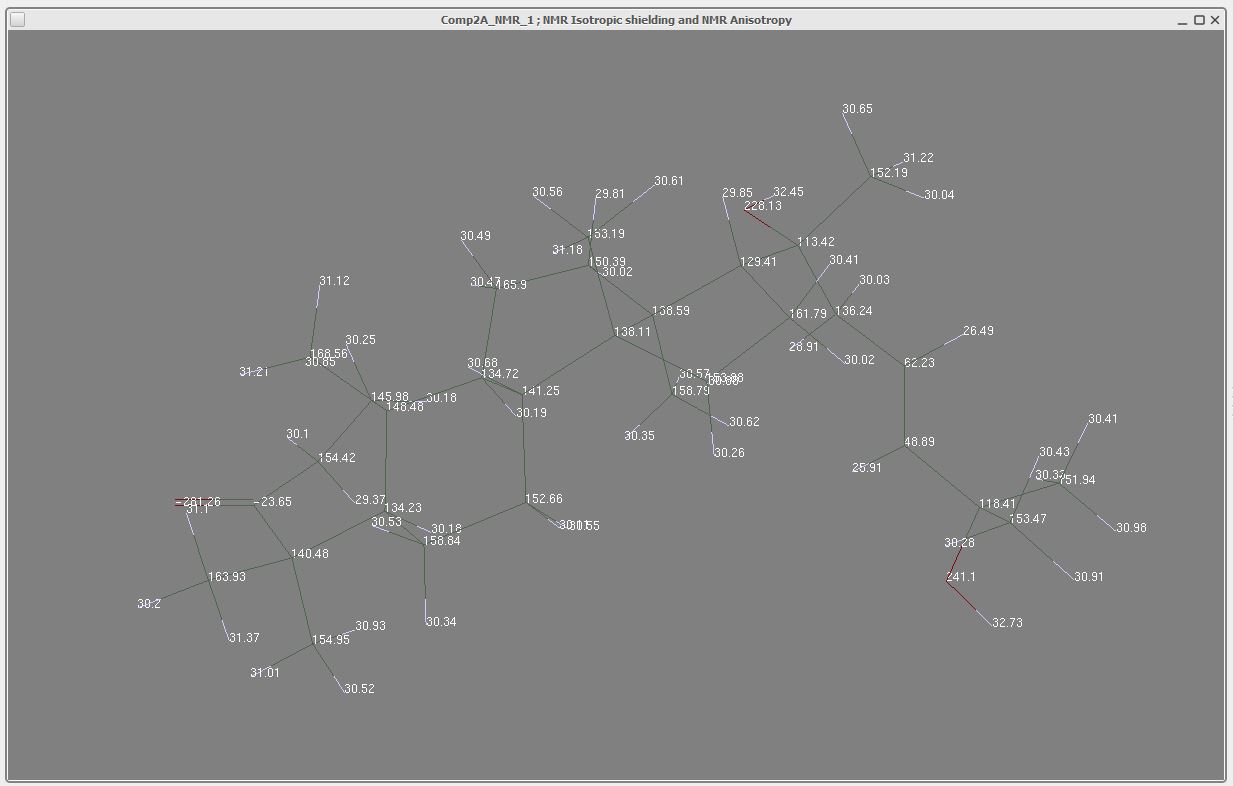


**Figure S39.** Optimized ground state structure and NMR shielding constants for chemical shift calculation of isomer 2 of compound **2**.


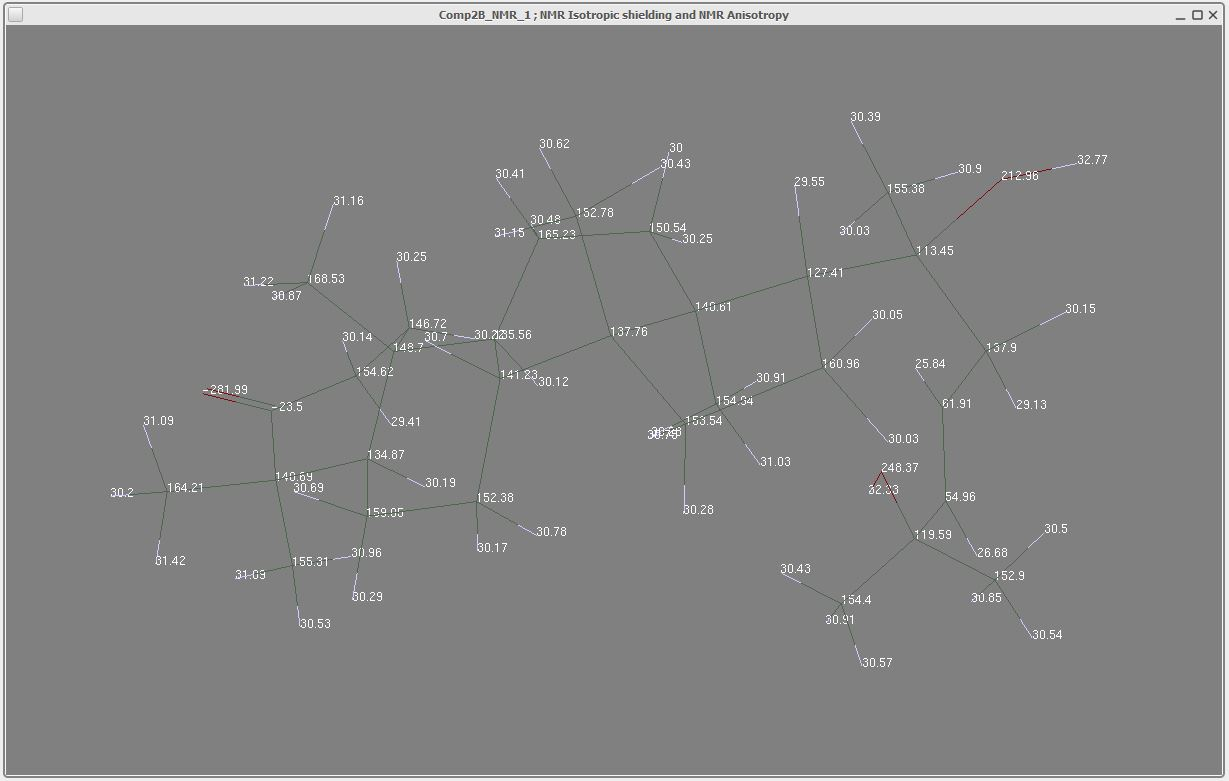


**Figure S40.** Optimized ground state structure and NMR shielding constants for chemical shift calculation of isomer 1 of compound **3**.


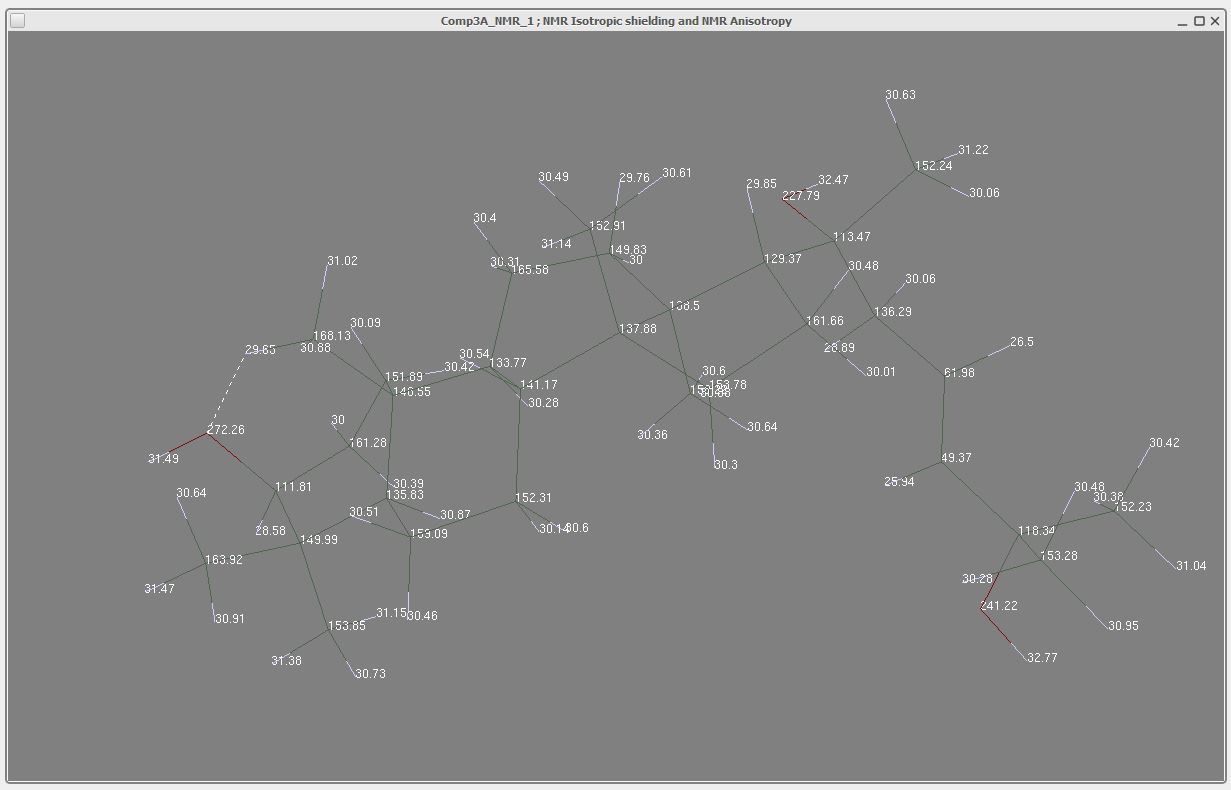


**Figure S41.** Optimized ground state structure and NMR shielding constants for chemical shift calculation of isomer 2 of compound **3**.


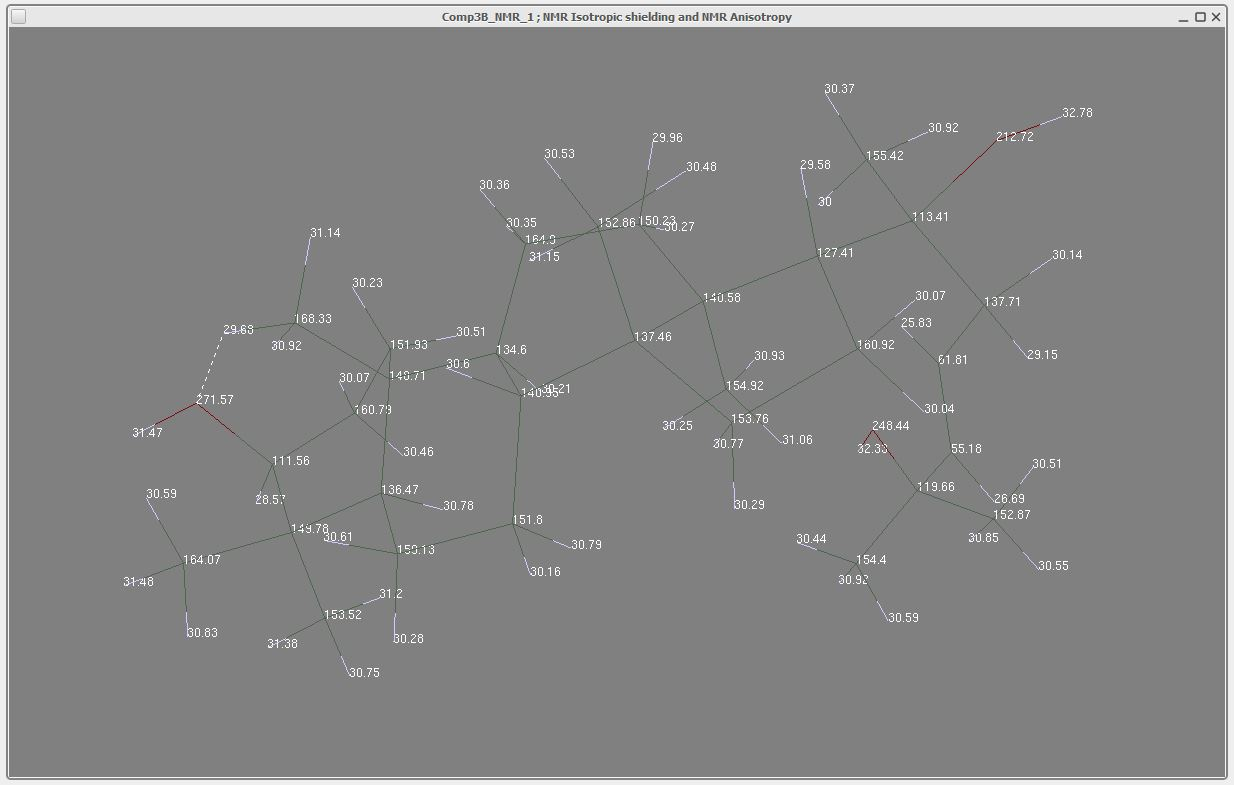

Supplement: Supplementary file 1 [file molecules-23-02732-s001.doc]
